# Diverse Marine T4-like Cyanophage Communities Are Primarily Comprised of Low-Abundance Species Including Species with Distinct Seasonal, Persistent, Occasional, or Sporadic Dynamics

**DOI:** 10.3390/v15020581

**Published:** 2023-02-20

**Authors:** Emily Dart, Jed A. Fuhrman, Nathan A. Ahlgren

**Affiliations:** 1 Department of Biology, Clark University, Worcester, MA 01610, USA; 2 Department of Biological Sciences, University of Southern California, Los Angeles, CA 90089, USA

**Keywords:** phage, cyanobacteria, viromics, phage–host interactions, microbial ecology, marine, oceanography

## Abstract

Cyanophages exert important top-down controls on their cyanobacteria hosts; however, concurrent analysis of both phage and host populations is needed to better assess phage–host interaction models. We analyzed picocyanobacteria *Prochlorococcus* and *Synechococcus* and T4-like cyanophage communities in Pacific Ocean surface waters using five years of monthly viral and cellular fraction metagenomes. Cyanophage communities contained thousands of mostly low-abundance (<2% relative abundance) species with varying temporal dynamics, categorized as seasonally recurring or non-seasonal and occurring persistently, occasionally, or sporadically (detected in ≥85%, 15-85%, or <15% of samples, respectively). Viromes contained mostly seasonal and persistent phages (~40% each), while cellular fraction metagenomes had mostly sporadic species (~50%), reflecting that these sample sets capture different steps of the infection cycle—virions from prior infections or within currently infected cells, respectively. Two groups of seasonal phages correlated to *Synechococcus* or *Prochlorococcus* were abundant in spring/summer or fall/winter, respectively. Cyanophages likely have a strong influence on the host community structure, as their communities explained up to 32% of host community variation. These results support how both seasonally recurrent and apparent stochastic processes, likely determined by host availability and different host-range strategies among phages, are critical to phage–host interactions and dynamics, consistent with both the Kill-the-Winner and the Bank models.

## 1. Introduction

T4-like cyanophages represent one of the most abundant groups of viruses in the oceans and have important impacts on the mortality, productivity, and evolution of their globally significant host populations, *Prochlorococcus* and *Synechococcus* [1,2,3]. Interactions between host and viral communities are complex, with viruses exerting top-down controls on host populations while, in turn, having their own abundances influenced by the seasonal and annual variation of bacterial host abundances [4,5]. Elucidating these complex virus–host interactions is critical to our fundamental understanding of how viral and microbial diversity and community structure are maintained in natural ecosystems. T4-like cyanophages represent one of the most extensively studied groups of marine viruses, and here we carry out quantitative analyses of their community structure and dynamics in combination with host community analysis.

Viral population dynamics are most frequently described using two non-mutually exclusive models: the Kill-the-Winner model (KtW) and the Bank model. KtW posits that due to density-dependent effects, as permissive bacterial hosts become abundant, viruses that are able to infect them will also rise in abundance and decimate permissive host populations, thus allowing resistant hosts to increase in abundance [6,7]. This model predicts dynamic cycling and maintenance of multiple host and virus variants. Studies suggest that this cycling occurs more so at fine scales of diversity, e.g., at the level of species or strains, while communities may remain relatively stable at higher taxonomic levels, e.g., ecotypes [8,9,10,11]. The Bank model suggests that most viral variants remain at low abundance, only becoming abundant in the presence of their preferred, susceptible host(s) [5,12,13,14,15]. 

Because phage abundances are dynamic across seasons, the use of time series to study natural phage communities has proven to be a valuable approach to studying phage–host interactions [10,16,17]. T4-like cyanophages have been extensively studied using several informative, although not entirely cyanophage-specific, marker genes, including the major capsid protein, *gp23*, and the vertex portal protein, *gp20*. Marker gene analyses show clear temporal variation in phage communities across a variety of locations, indicating that cyanophage communities are driven by host presence and/or environmental variables [13,18,19,20,21,22]. While whole T4-like phage communities show seasonality, multiple studies have identified individual phage operational taxonomic units (OTUs) that show a variety of annual and interannual dynamics, including OTUs that persist in environments over multiple years and OTUs that appear only briefly in a system [21,22,23]. These different patterns are interpretable under the KtW or Bank models and may reflect underlying differences in phage–host range as observed from culture studies—some phages appear to be specialists and infect a narrow range of hosts, while others are more generalists and infect multiple hosts, even across genera [1,24,25].

Although T4-like cyanophage populations have been widely studied [10,13,16,18,19,20,21,22,23,24], at present, there are relatively few studies that have tracked the dynamics of viral populations with sustained regular sampling and with appropriate quantitative methods to track fine-scale variants. In particular, the studies above are limited in that they used clone libraries [23] or viral isolates [18,19,22,24] that are semi-quantitative and sampled a limited number of phages; terminal restriction fragment length polymorphism (tRFLP) methods that lack phylogenetic information about OTUs [10,20]; or, for studies using metagenomics or high-depth amplicon sequencing, did not sample phage populations for more than two years [13,16,21]. Phylogenetic context is particularly important for resolving biologically relevant taxa, namely, species-like populations. Furthermore, most of the studies above lack concurrent analysis of host community dynamics.

The majority of prior work that has concurrently studied both T4-like phage communities and cyanobacteria communities has been carried out in the surface layer of the coastal, temperate waters at the San Pedro Ocean time series (SPOT), located in the San Pedro Channel, California, USA. Seasonal dynamics of cyanobacteria populations at SPOT have been well studied. Broadly, *Synechococcus* are more abundant in warmer months, peaking in April through June with a community dominated by a single ecotype, IV, and seasonal succession seen in subclades of ecotype I and pulses of less abundant ecotypes [10]. *Prochlorococcus,* in contrast, increases in abundance during the winter months and is dominated by a single ecotype, HLI [10]. These seasonal changes in the host community likely impact changes within the viral community. Network analyses at SPOT using *gp23* tRFLP data show significant correlations between host and viral OTUs indicating that the seasonal dynamics seen in phage communities are driven in part by the seasonal changes of preferred hosts [10,11,26]. However, the most robust of these concurrent virus–host analyses [10] could not reliably determine the identity or relatedness of phage populations, namely those of cyanophages, due to the use of tRFLP methods rather than sequence-based methods and the lack of resolution provided by the *gp23* marker gene. In addition to missing fine-level resolution of T4-like cyanophage dynamics, critical fundamental ecological descriptions have not been performed for T4-like cyanophages, such as rank abundance analysis or estimates of richness for biologically relevant taxonomic groups, namely species. Recent work has shown that T4-like cyanophage communities form stable species-like populations that share roughly >95% average nucleotide identity (ANI) across shared genes [24,27]. We refer to populations matching this threshold as species throughout this work.

Focusing on just T4-like cyanophages, here we resolve species populations using a 98% nucleotide identity threshold in the DNA polymerase *gp43* amplicon region corresponding to distinct populations as guided by ANI differences across shared genes in cultured cyanophages as described in Gregory et al. 2016 [24]. We use *gp43* cyanophage sequences found in cellular and viral fraction metagenomes in combination with cyanobacterial ITS amplicon data to study the dynamics of T4-like cyanophage species and their hosts at the San Pedro Ocean Time-series across five years of monthly samples. These results reveal highly diverse phage communities with complex dynamic patterns among phage species, significant correlations to *Prochlorococcus* and *Synechococcus* communities, and distinct patterns between cellular and viral fraction samples. Together these results refine our understanding of phage community structure and dynamics, especially in the context of current phage–host interaction models.

## 2. Materials and Methods

### 2.1. ANI-Informed Classification of Cyanophage Species

The map primer function in Geneious Prime (v.2019.2.3. Biomatters Ltd., Auckland, New Zealand) was used to align the gp43For-5′GCWGGTGCWTATGTHAARGAACC-3′ and gp43REV-5′CCWGASARAGTAATKGCYTCWGC-3′ primers [28] to the 142 cyanophage isolate genomes described in Gregory et al. 2016 [24] to identify the *gp43* amplicon region. Genomes from Group VI [24] have a large insertion of ~25–50 bases 3′ of the gp43For primer, so this insertion region was removed to retain homologous comparisons of the *gp43* amplicon region to the other genomes. The nucleotide identity of corresponding *gp43* amplicon regions was calculated using the blastn function of BLAST (v. 2.2.29+). For all pairwise combinations, *gp43* nucleotide identity was compared to the ANI across the shared core genes identified in Gregory et al. 2016 [24].

### 2.2. Sample Collection and Processing

Seawater for DNA samples, as well as measurements of biological and environmental conditions, were collected monthly as part of the San Pedro Ocean Time-series (https://dornsife.usc.edu/spot/ accessed on 10 January 2023). Here we use monthly samples collected between May 2009 and September 2014. Detailed methods describing the collection and processing of environmental and biological parameters for these samples are described Cram et al. 2015 [29]. Briefly, for the collection of DNA samples from cellular and viral fractions, water was filtered sequentially through a 1.2 µm A/E filter (Pall, Port Washington, NY, USA) and then a 0.2 µm Durapore filter (EMD Millipore, Billerica, MA, USA) [30] to collect DNA primarily from microbial cells, referred to herein as the cellular size fraction [10,29]. Viral fraction DNA was obtained by filtering water through a 0.22 µm Sterivex cartridge (EMD Millipore, Billerica, MA, USA) and then onto a 25 mm 0.02 µm Anotop filter cartridge (Cytiva, Marlborough, MA, USA) [10,11]. Sample dates and sample type collected on each date are provided in Table S1.

Cellular metagenomes were prepared with 40 ng of genomic DNA from the cellular fraction. DNA was sheared to 500 bp, library preparation was carried out using the Ovation Ultralow V2 DNA-Seq kit (Tecan Group Ltd., Männedorf, Switzerland), and libraries were sequenced on a HiSeq 2500 Rapid Run PE 100 or 250. Viral fraction metagenomes were generated and sequenced at the Department of Energy (DOE) Joint Genome Institute (JGI) as part of the Community Science Program on a grant to N.A.A. (proposal ID, 2799). Libraries were prepared using 1 ng of viral fraction DNA per sample and according to the manufacturer’s instructions (Swift 1S Plus or Nextera XT; details for individual samples can be found under proposal 2799 at the JGI Genome Portal) [11].

The abundance of picocyanobacterial (*Prochlorococcus* and *Synechococcus*) amplicon sequence variants (ASVs) was determined using ITS amplicon sequencing from the same DNA samples as were used to produce the cellular metagenomes. ITS amplicon sequencing details have been previously published and can be found in Ahlgren et al. 2019 [10]. Briefly, ITS amplicon sequences were amplified from the cellular fraction DNA samples using the 16S-1247f [31] and ITS-ar [32] primers. Unique amplicon sequence variants (ASVs) were identified using minimum entropy decomposition [33] and are sequence variants that differ by at least one nucleotide difference from any other variant. ASVs were assigned to ecotypes using blastn to a database of full-length ITS sequences [10]. Ecotypes have been previously well established as phylogenetically distinct groups that inhabit distinct niches based on temperature and nutrient conditions and are distinguishable using ITS sequencing [34,35,36].

### 2.3. Assembly and Identification of gp43 Sequences

Viral metagenomes were assembled by the Joint Genome Institute as described in Ignacio-Espinoza et al. 2020 [11]. In brief, reads were trimmed and screened using bbtools [37] before being error-corrected using bfc (v.r.181) [38]. Paired reads were assembled using SPAdes (v.3.11.1) [39]. Raw and quality-controlled reads, as well as virome assemblies, can be found on the DOE JGI Genome portal under proposal ID 2799. Cellular metagenomes were assembled using MEGAHIT (v1.1.3) [40] using default parameters. Contigs <1000 bps were discarded from both virome and cellular metagenome assemblies. Viral contig prediction was carried out using both VirSorter (v1.0.5) [41] and VirFinder (v1.1) [42]. VirSorter predictions were made using the virome database, and only contigs in categories 1 and 2 were retained. Contigs with VirFinder scores greater than 0.80 were used in subsequent analyses. To identify *gp43* sequences on assembled contigs, *gp43* sequences from 318 myovirus cyanophage genomes and 200 amplicon sequences from environmental isolates (Table S2) were used to compile a BLAST database. tBLASTn (v. 2.2.29+) [43] was used to compare viral contigs to the *gp43* database. Sequences sharing at least 60% amino acid identity over 67% of the length of one of the reference sequences were categorized as cyanophage *gp43* sequences. Only the *gp43* genes from contigs were used in subsequent analyses. 

Additionally, in order to capture a larger portion of the cyanophage diversity and avoid the long contig biases of VirSorter and VirFinder [41,42], single-gene assembly of the *gp43* amplicon region was also performed using Xander [44] with the following changes to the default settings: MIN_COUNT = 1 and MIN_LENGTH = 100. *gp43* sequences from reference cyanophage genomes and environmental amplicons were used to construct the Xander gene seed and gene reference fasta files.

### 2.4. Clustering and Mapping 

All assembled and reference *gp43* sequences were de-replicated using the derep_fulllength of Vsearch (v2.15.2) [45] with default parameters and clustered to 98% nucleotide sequence identity using the cluster_fast function. BBmap (v38.08) [37] was used for mapping of short reads from viral and cellular metagenomes to the *gp43* sequences and requiring ≥98% sequence identity. Only *gp43* sequences with 100% base coverage and ≥2-fold depth in at least one sample were used for future analyses. Throughout, the relative abundance of species in a sample was calculated as reads mapped per kilobase per million reads (RPKM).

### 2.5. Cyanophage Community Analysis 

Total estimated species richness using the Chao estimate was determined using the function specpool in the R package vegan [46] using mapped read counts to *gp43* sequences from all samples as the input. Rarefaction curves of individual samples were generated with the function rarecurve in the package vegan. Bray–Curtis dissimilarity scores were calculated using the d_bray command from the R package phyloseq (v. 1.34.0) [47] for all possible pairs of samples and results were binned by the number of months separating samples. Bray–Curtis dissimilarity values were subtracted from one to convert to a community similarity value. Because samples separated by 1–2 months had average Bray–Curtis values that were much greater than Bray–Curtis values for samples separated by ≥3 months, sinusoidal curve fitting on Bray–Curtis values was performed for samples with 3–36 months separating them using the lm function from the R Stats Package (v. 4.0.3) [48].

### 2.6. Seasonal Dynamics 

Each *gp43* sequence and host ASV was tested for interannual and seasonal variability using a generalized additive mixed-effects model (GAMM) using the R package gamm4 [49,50,51]. *gp43* sequences were tested for seasonality in both the cellular and viral metagenomes separately. The data were fit using a seasonally cyclic spline and a spline that fit the overall data as per Cram et al. 2015 [29]. The *p*-values for both spline functions were used to determine whether phage OTU exhibited seasonality (i.e., those for which the seasonal component had a *p*-value of <0.05 were categorized as seasonal phages). This approach for determining seasonality detects non-sinusoidal seasonal patterns as well as seasonal patterns that have strong interannual variation [49,52]. Raw *p*-values, excluding those with a value of one, were adjusted with the R function qvalue to account for false discovery rates [53].

To assess the potential impact of differences in library size and coverage between size fractions on seasonal categorization, read counts to *gp43* species in the viral fraction metagenomes were divided by five (the mean fold difference in the percentage of reads that mapped to *gp43* in the viral vs. cellular fraction samples). Any resulting read counts below 1 were reassigned as 0 values, and RPKM was recalculated. The model as described above was rerun as above to assign dynamic types.

### 2.7. Network Analysis 

Significant correlations were identified using extended local similarity analysis (eLSA v1.0.2) [54] among environmental parameters (*n* = 45); *gp43* species (*n* = 418) abundances (RPKM) in both the viral and cellular fraction metagenomes; absolute cyanobacterial ecotype abundances; and absolute cyanobacterial ASV abundances for those that comprised over 0.5% of the host population in at least one sample (*n* = 300). eLSA was run with the following parameters -d 3 -p theo -r 1 -s 32 -b 0. Sample dates for months between December 2009 and December 2013 were used for this analysis (see Table S1 for months in this range during which data were collected). Because eLSA can accommodate missing data, months for which no data were available or for which only host or viral data were available were included with missing data input as NA. Environmental parameters included the following: month, maximum air temperature, minimum air temperature, average wind speed, precipitation, wind speed, prokaryotic abundance, average wave period, Multivariate El-Niño Southern Oscillation Index (MEI), dominant wave period, wave height, monthly estimated primary productivity, absorbance due to detritus and gelbstoff, absolute phytoplankton abundance, satellite-based chlorophyll *a*, chlorophyll *a* maximum depth, day length, elapsed days, leucine production, mixed layer depth, nitrite concentration, nitrate concentration, oxygen concentration, oxygen saturation, PAR, phosphate concentration, particulate organic carbon, primary production, excess phosphate concentration (P*), eight day average estimates for surface chlorophyll *a* concentration and surface productivity, salinity, Sigma-theta, Pacific Fisheries and Environmental Laboratory (PFEL) estimates of coastal upwelling and Sverdrup transport (SVD), temperature, thymine, leucine turnover, thymine turnover, virus-to-prokaryotic ratio (VPR), total virus abundance, and flow cytometer *Synechococcus* and *Prochlorococcus* concentrations (see Cram et al. 2015 for details [29]). Results were filtered to only include significant (*p* < 0.0015 and Q < 0.05) and strong Spearman correlations (*p* ≥ 0.6). Network visualization was performed using Cytoscape (v. 3.8.0) [55]. Small subnetworks containing fewer than five nodes were omitted.

### 2.8. Phylogenetic Analysis 

*gp43* sequences were aligned using transAlign (v. 1.2) [56]. Phylogenetic tree construction was carried out using RaxML v8 [57] with the GTRCAT substitution model using rapid bootstrapping on CIPRES infrastructure [58]. A subset of eleven reference T4 cyanomyovirus *gp43* sequences was included, and the tree was rooted using an *Escherichia coli* T4 phage sequence (NCBI accession MT984581).

### 2.9. Variance Partitioning 

Variance partitioning analysis was performed using redundancy analysis (RDA) and partial RDA [59] to assess how much variance in cyanobacterial community composition can be explained by concurrent variance in environmental factors and cyanophage community composition and the relative, independent contribution of either. The analyses followed those outlined in Ahlgren et al. 2019 [10] and are described in brief as follows. All environmental parameters used previously in Ahlgren et al. 2019 [10] were used. This includes: month, average wind speed, precipitation, prokaryotic abundance, average wave period, dominant wave period, wave height, absolute phytoplankton abundance, satellite-based chlorophyll *a*, chlorophyll *a* maximum depth, day length, elapsed days, leucine production, mixed layer depth, nitrite concentration, nitrate concentration, phosphate concentration, excess phosphate concentration (P*), oxygen concentration, salinity, temperature, leucine turnover, virus-to-prokaryotic ratio (VPR), total virus abundance, Pacific Fisheries and Environmental Laboratory (PFEL) estimates of coastal upwelling and Sverdrup transport (SVD) (see Cram et al. 2015 for details [29]). The function decostand from the R package vegan was used to first standardize environmental data to zero mean and unit variance, and Hellinger transformation was applied to the relative abundances of cyanophage species and the absolute abundances of cyanobacterial ecotypes and ASVs. Absolute cyanobacteria abundances were calculated by multiplying appropriate relative abundances from ITS sequence data by total *Prochlorococcus* or *Synechococcus* concentrations determined by flow cytometry [10]. Data were reduced using a forward selection of principal component axes and the function *ordistep* for added axes where *p* < 0.1. The function *rda* was used to perform RDA and partial RDA analyses, with the significance of results assessed by ANOVA using 200 steps and up to 200 permutations. The function *varpart* was used to determine the unique fraction of variance that cyanophage composition and environmental parameters contributed to the variance in cyanobacterial community composition. RDA and partial RDA were repeated for three different levels of diversity for the cyanobacterial (host) communities: all host ecotypes, *Prochlorococcus* ecotypes only, *Synechococcus* ecotypes only, all host ASVs, *Prochlorococcus* ASVs only, and *Synechococcus* ASVs only.

## 3. Results

### 3.1. Classification of T4-like Cyanophage Species 

*gp43* is a core gene found in all T4-like cyanophage to date, and the PCR amplicon region in this gene has previously been used to identify emergent, distinct populations—namely, populations comprised of individuals that differ by <99% nucleotide identity [22]. To better relate *gp43* divergence to more recently proposed ANI-based methods for demarking T4-like cyanophage populations [27], we identified and compared the nucleotide identity of the *gp43* amplicon region to core gene ANI for 142 isolate genomes from Gregory et al. 2016 [24], representing six discrete lineages that can be considered distinct populations using a proposed ANI threshold of 95% [27]. As in Gregory et al. 2016, we saw a conspicuous gap in ANI between intra- and inter-species comparisons of >97% and <88%, respectively, and a corresponding gap in *gp43* identity at >99% and <90%, respectively (Figure S1). Such gaps [22,24,27] have been proposed to reflect inherent boundaries in populations [24,27] and informed the proposed 95% ANI threshold for classifying T4-like cyanophage populations [27]. To delineate cyanophage species here using *gp43*, we likewise chose a threshold of 98% that falls within the observed gap but is slightly lower than the previously used threshold of 99% [22], noting that some within species comparisons approached 99% *gp43* identity (Figure S1). This benchmarked threshold of 98% *gp43* nucleotide identity, therefore, was used to classify T4-like cyanophage species in metagenomically assembled *gp43* sequences. We used analysis of *gp43* cyanophage sequences in viral (<0.2 µm) and cellular fraction (0.2 to ~1.2 µm) metagenomes to elucidate the composition and dynamics of T4-like cyanophage species in surface waters at SPOT. *gp43* sequences were obtained from the assembly of cellular metagenomes (*n* = 2905) and viral metagenomes (*n* = 5518) using whole metagenome assembly (MEGAHIT and SPAdes, respectively) or focused single-gene assembly (Xander) (*n* = 6680). All assembled *gp43* sequences were clustered with reference isolate sequences (*n* = 288) with a threshold of 98% nucleotide similarity. This resulted in 3562 T4-like cyanophage species identified from both sets of the metagenomes. Only species with 100% coverage over the length of the *gp43* amplicon region and ≥2X read depth in at least one sample (*n* = 354) were used in subsequent analyses unless noted otherwise to eliminate low-coverage, spurious taxa. Of these species, only seven appeared in the viral fraction and not in the cellular fraction; all others were represented in both viral and cellular metagenomes. Notably, no reference cyanophage isolate *gp43* sequences clustered (i.e., had ≥98% nucleotide identity) with any metagenomic sequences, and no isolate reference sequences met the coverage required to be included in our analyses.

### 3.2. T4-like Cyanophage Community Composition and Whole Community Dynamics 

Total cyanophage species richness across all of the viral and cellular metagenomes separately was estimated to be 2988 ± 49.8 and 1851 ± 52.7 (±standard error), respectively, using the Chao1 estimator and read counts for all detected species (no coverage requirement). These estimated values were slightly higher than the total number of species found in the viral and cellular fraction metagenomes, 2566 and 1410, respectively, indicating that this approach appears to have sampled most of the cyanophage species present. Rarefaction curves of individual samples likewise showed a high degree of saturation (Figure S2A,B). Repeating these analyses when requiring the coverage and depth noted above yielded similar looking rarefaction curves but with somewhat higher levels of saturation Figure S2C,D). Chao1 estimates of total richness were 357 ± 3.4 and 357 ± 5.1, very similar to the total number of species detected: 354 and 347, respectively. The T4-like cyanophage community therefore likely contains thousands of species, most of which did not meet our read recruitment requirement for tracking their abundance.

**Figure 1:**
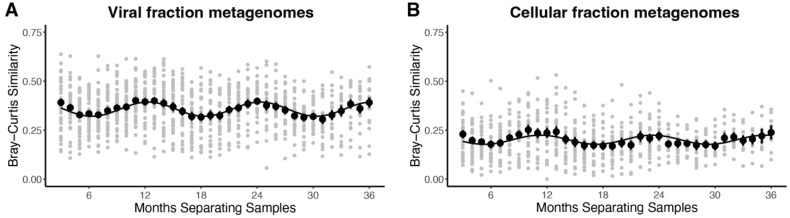
Seasonality of whole T4-like cyanophage community is evidenced by Bray–Curtis (BC) similarity scores (gray points) of cyanophage communities as a function of months separating samples. Black points show average BC similarity values. Errors bars are standard error. The black line is a sine fit to average BC values. (**A**) T4-like cyanophages found in viral fraction metagenomes. *p*-value for sine fit = 9.90 × 10^−13^. (**B**) T4-like cyanophages found in cellular fraction metagenomes. *p*-value for sine fit = 2.20 × 10^−5^.

The total abundance of cyanophage *gp43* species relative to the total numbers of reads in viral metagenomes was markedly stable over the time series (Figure S3), which mirrors the pattern that total virus-like particle counts from this location do not vary widely by season [29]. However, analysis of Bray–Curtis community similarity values (1 – Bray-Curtis dissimilarity scores) revealed that cyanophage community composition showed marked seasonality, as seen from significant sine curve fitting of similarity values vs. the number of months separating samples (Figure 1) and local maxima and minima in similarity values at or near 12, 24, and 36 months and 6, 18, and 30 months, respectively. Cyanophages in the viral fraction, however, had higher community similarity scores on average than those for the cellular metagenomes for samples separated by one month and 12, 24, and 36 months near local maxima (*t*-test, *p* < 0.001) (Figure 1).

### 3.3. Dynamic Patterns of Individual Cyanophage Species 

**Figure 2:**
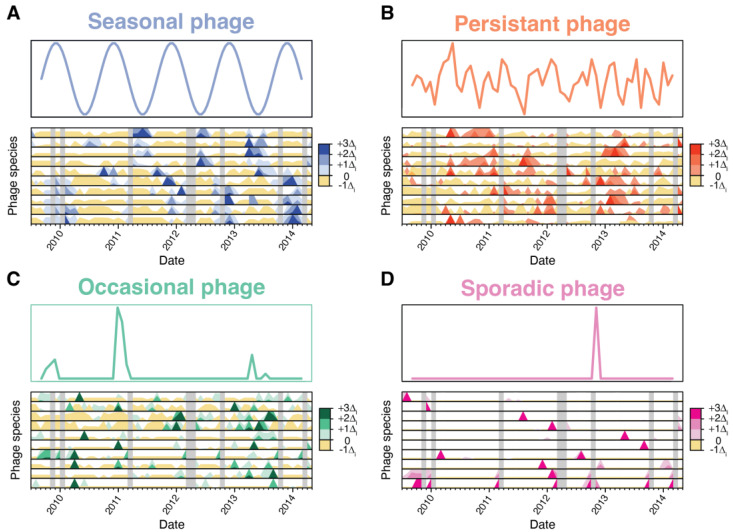
T4-like cyanophages belong to one of several dynamic classes. Horizon plots (lower plot in each panel) show select representative cyanophage species belonging to the (**A**) seasonal, (**B**) persistent, (**C**) occasional, and (**D**) sporadic dynamic categories (see text for descriptions). The top plot in each panel depicts idealized representations of the dynamics of each phage dynamic category. For horizon plots, the relative abundance of each phage species (one species per row) is centered around the mean abundance, with the intensity of color indicating changes in abundance by standard deviations above or below the mean. Gray vertical bars indicate months with missing data.

Although the collective cyanophage community showed significant seasonality in both cellular and viral fractions, individual viral species showed a variety of distinct dynamics. Using a non-parametric regression model with seasonal and long-term splines, phage species were classified into four classes: seasonal (seasonal spline *p*-value < 0.05), persistent (the species was detected >85% of samples regardless of seasonal spline see Table S3), sporadic (not seasonal and detected in <15% of samples), and occasional if they fit none of the above classes (Figure 2).

**Figure 3:**
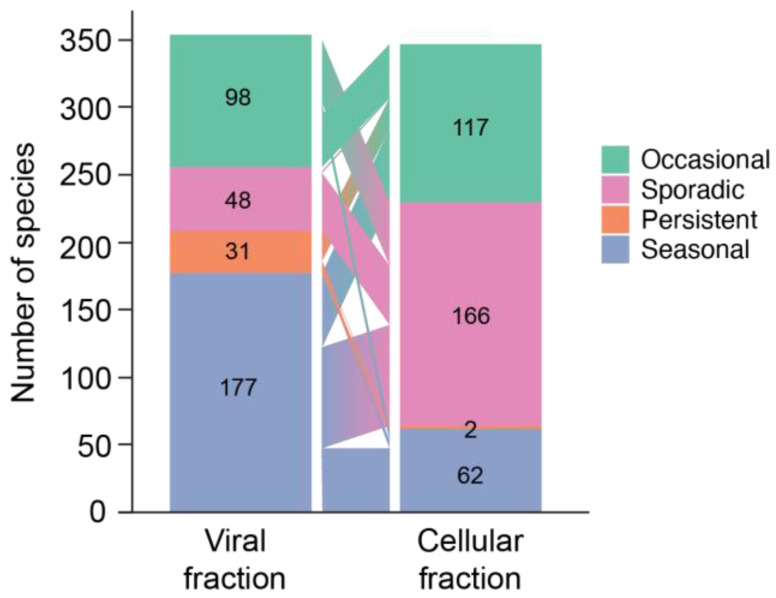
The number of species categorized to each dynamic class (seasonal, persistent, occasional, and sporadic) in the viral and cellular fraction metagenomes (left and right bars, respectively) and an accounting of how each species is categorized differently between each sample type (trapezoids in the middle). Note that seven species detected in the viral fraction metagenomes were not detected in the cellular fraction metagenomes.

**Figure 4:**
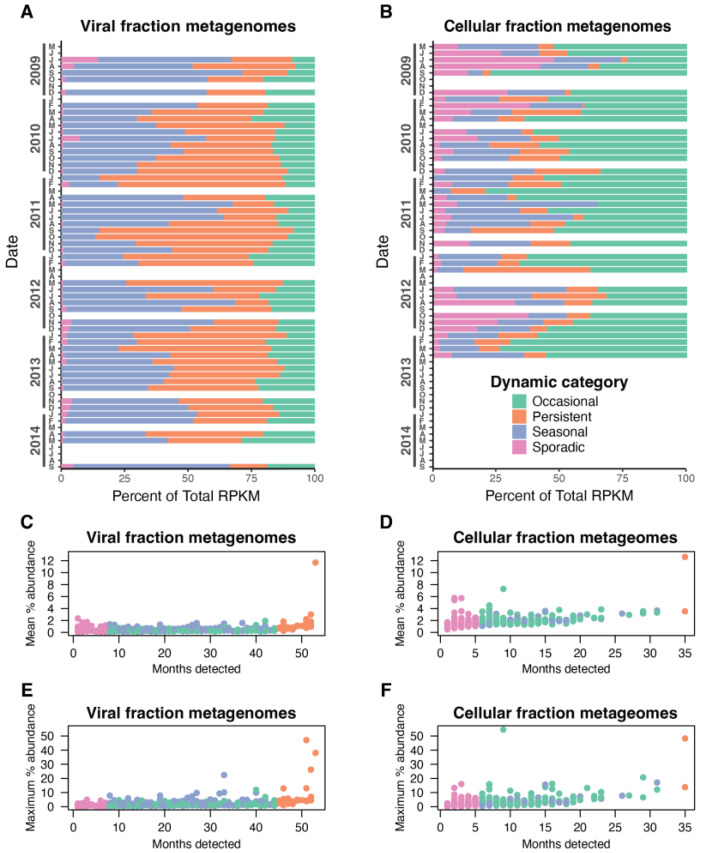
The contribution of species to the T4-like cyanophage community. (**A**,**B**) Cumulative abundances of cyanophages in each dynamic class in viral (<0.2 µm) (**A**) and cellular fraction metagenomes (0.2–1.2 µm) (**B**) as determined by read mapping (RPKM). Results are shown sequentially for each month, with blank lines indicating no data for that month. (**C**,**D**) The average percent contribution of each cyanophage species across all time points when present vs. the number of times that viral species was detected (“Months detected”) in (**C**) viral and (**D**) cellular fraction metagenomes. (**E**,**F**) The maximum percent abundance of each species relative to total T4-like cyanophage abundance across all viral (**E**) and cellular (**F**) fraction metagenomes. In (**C**–**F**), each point represents a distinct cyanophage species.

The classifications above were determined for cellular and viral size fractions separately. Phage species were accordingly given two classifications—one from the cellular fraction and one from the viral fraction. The composition of species in each of these classes differed significantly between the cellular and viral fraction metagenomes (*p*-value < 0.05, chi-squared test). This difference was particularly marked for seasonal and sporadic species. About half of the species in the cellular metagenomes were classified as sporadic, whereas about half of the phages in the viral metagenomes are seasonal (Figure 3, see also Figure 4). While there were several persistent phages in the virome, notably, only two phage species were categorized as persistent in the cellular metagenomes. The classification of individual viral species correspondingly often differed between the viral and cellular fraction metagenomes (Figure 3), and there were only seven species (three sporadic and four occasional) that were detected in the viral fraction but not in the cellular fraction samples. At any given time point, many species were detected in both the viral and cellular fraction metagenomes: on average, 40% of species present in the viral fraction and 84% of the species present in the cellular fraction were detected in the sample from the other size fraction (Table S1). To assess whether differences in temporal categorization between size fractions might be attributed to differences in library size, we simulated a 5-fold reduction in the size of viral fraction metagenomes and reassigned dynamic classes. The resulting composition of dynamic classes in the downsampled virome was quite similar to the original categorization (Figure S4), and differences between dynamic classes in the cellular and the downsampled viral metagenomes remained significant (*p*-value < 0.05, chi-squared test) (Figure S4).

The cumulative relative abundance of species belonging to these categories was relatively stable over time in both metagenome fractions (Figure 4A,B) but, similar to the patterns above, differed between viral and cellular fraction metagenomes. Each dynamic group again differed significantly between the two metagenome fractions (chi-squared test, *p* < 0.001). Viral fraction metagenomes were composed mainly of persistent and seasonal cyanophages (40% and 41% on average, respectively), while cyanophages in cellular metagenomes instead were dominated mostly by occasional cyanophages (50% on average) (Figure 4B). Sporadic cyanophages only comprised a very small proportion of species in viral fraction metagenomes on average (1.6%) but were much more abundant in cellular fraction genomes (10.4%) (Figure 4A,B).

**Figure 5:**
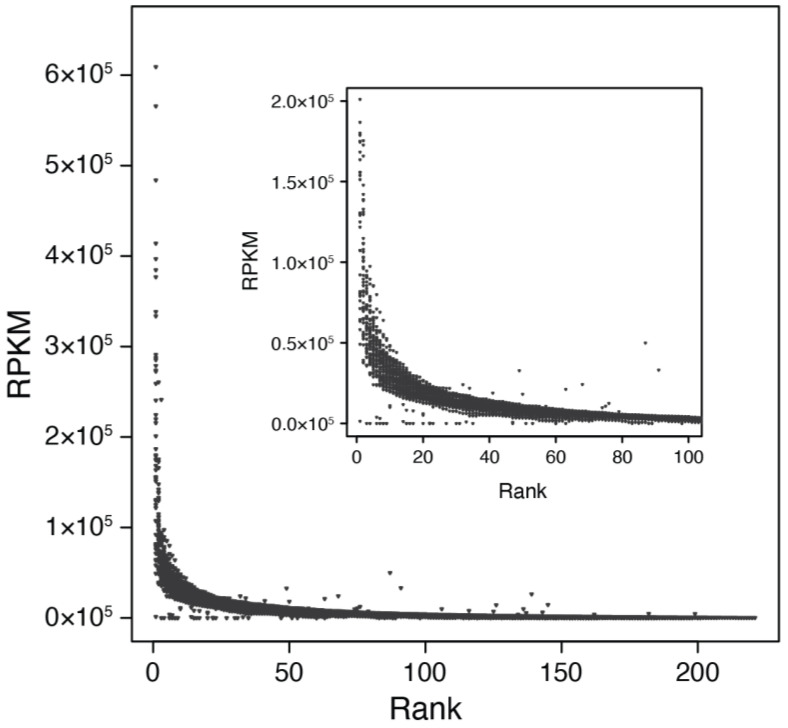
Rank abundance curve for all viral species across all viral fraction metagenomes. Insert shows ranks between 1 and 100. Viral species abundance depicted on the *y*-axis is read coverage (RPKM). The shape of this rank abundance curve is typical of microbial communities with a few very abundant species in any given sample and numerous less abundant species.

The relative abundance of individual species revealed that T4-like cyanophage communities are primarily composed of many low-abundance species with only one species having a high mean relative abundance in both fractions at roughly ~12% (Figure 4C,D). A few species in the cellular metagenomes had notably higher mean abundances at ≥5%, and several species in the viral metagenomes had abundances ≥ 2% across all dynamic types (Figure 4C,D). In terms of maximum abundance, some species ‘bloomed’ to substantial levels at particular times, with several reaching > 10% and one species in each sample set comprising ~ 50% of the total T4-like cyanophage abundance (Figure 4E,F). Each species had similar abundance levels in the viral and cellular fraction metagenomes, as supported by strong and significant correlations of RPKM or relative abundance between the virome and cellular metagenomes (Figure S5, *p* < 0.001, R^2^ = 0.63 and 0.63, respectively). Mean and maximum relative abundances of species similarity were strongly correlated between the viral and cellular fraction metagenomes (*p* < 0.001, R^2^ = 0.56 and 0.67, respectively, Figure S5). These results collectively show that cyanophage communities contain several somewhat abundant species, but overall, the majority of the community is made up of species found at low average relative abundance (typically 1–2% of the community) (Figure 4C,D) with occasional higher maximum abundances (Figure 4E,F). This pattern is also reflected in a steep rank abundance curve with a long ‘tail’ of low-abundance species, typical of diverse marine bacteria, archaeal, and viral communities (Figure 5). Likewise, the richness estimates above suggest that there are thousands of T4-like cyanophage species, most of which were too rare to reasonably measure their abundance using this metagenomic approach.

### 3.4. Community Network Analysis 

**Figure 6:**
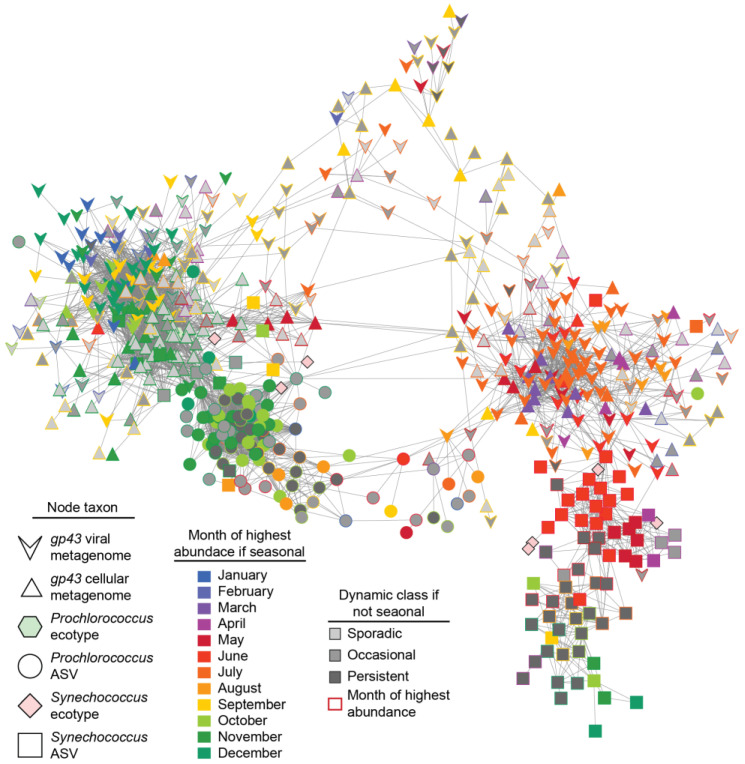
Network analysis of cyanophages and cyanobacteria. The network depicts correlations among viral species in viral fraction and cellular fraction metagenomes, host ASVs, and ecotypes as determined by eLSA with only strong and significant (Q < 0.05, ρ ≥ 0.6, Spearman correlation) interactions shown. The fill color of phage species indicates the dynamic class to which they belong, and if seasonal, the color indicates the month that they are predicted to be most abundant by the GAMM. For other dynamic classes (sporadic, persistent, or occasional), the outline color indicates the month at which they were most abundant.

To assess interactions among viruses, hosts, and environmental conditions, we examined correlations among viral species abundances in both the viral and cellular fraction metagenomes (RPKM), cyanobacterial ecotype and ASV abundances, and environmental parameters. Extended local similarity analysis (eLSA), which accounts for time-delayed relationships, was used to identify correlated viral species and host ASVs; only strong, significant relationships (Q < 0.05, *p* ≥ 0.6, Spearman correlation) were examined. Such correlation and network analysis can help investigate patterns of community dynamics and interactions, including possible phage–host interactions. The network showed a high level of interconnectedness between viral species in the virome and cellular metagenomes (Figure 6), consistent with the fact that abundances of species in the virome and cellular metagenomes were strongly correlated to each other (Figure S5). The number of virus–virus and virus–host correlations between viral populations from the virome and cellular metagenomes were significantly different from one another (*p*-value < 0.05 chi-squared test), with the majority of virus–host correlations found in the cellular metagenome (Table 1).

Network analysis also revealed two emergent subnetworks of viral species with inverse seasonality that include connections to viruses based on their abundance in viral and cellular metagenomes. One subnetwork contained species that were more abundant from November to March (nodes predominantly filled with cool colors at the left of the network), while the other contained species that were most abundant from May to August (nodes mostly filled with warmer colors at the right of the network) (Figure 6). These two subnetworks of viral species were most often correlated to *Prochlorococcus* and *Synechococcus* ASVs, respectively, consistent with the fact that *Prochlorococcus* and *Synechococcus* at SPOT peak in abundance in fall to winter and spring to early summer, respectively [10,60,61]. While the majority of correlations were either host–host or virus–virus connections, there were subnetworks with many connections between phage and host ASVs, especially among ASVs belonging to the *Prochlorococcus* HLI ecotype (Figure 6). Although environmental parameters were included in the network analysis, day length was the only environmental parameter significantly correlated with multiple viral species; all others had at most a single significant correlation with a viral species.

### 3.5. Phylogenetic Relatedness of Cyanophage Dynamic Phenotypes 

**Figure 7:**
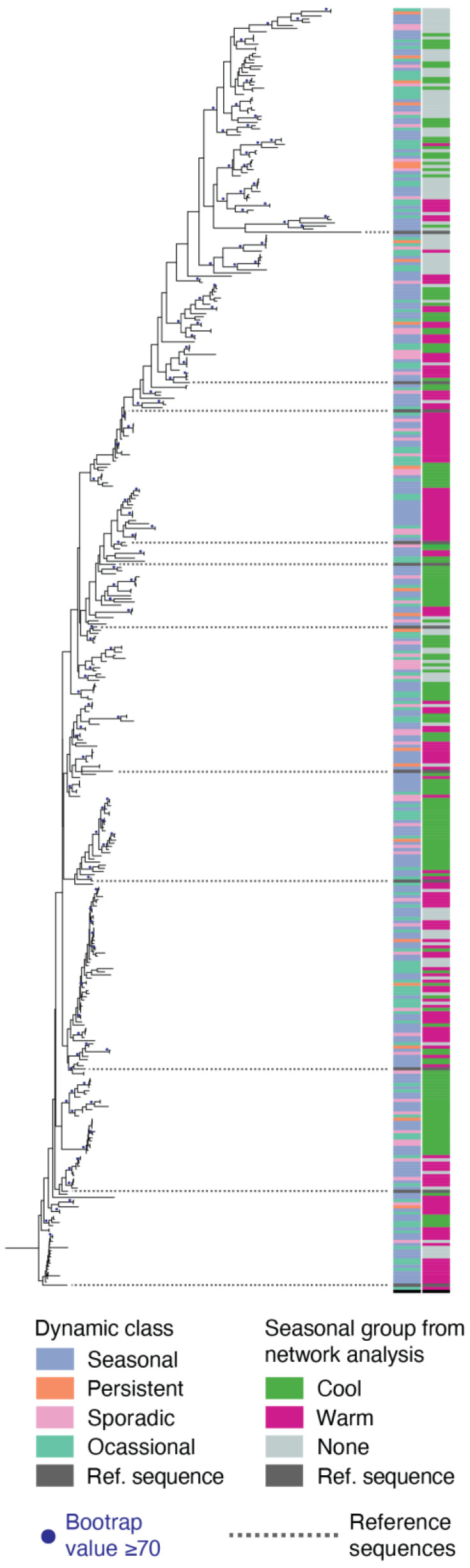
Maximum likelihood tree showing assembled *gp43* sequences and reference sequences. Tree is rooted using the *gp43* sequence for *E. coli* T4 phage. Colored blocks to the right of the tree indicate to which dynamic class (left column, e.g., see Figure 2) or seasonal group (right column; based on network analysis affiliation from Figure 6) each species belongs. Isolate *gp43* reference sequences, which were not detected in metagenomes, are indicated with dark gray blocks, and dashed lines show the location of these reference sequences in the tree.

Phylogenetic analysis showed that while some closely related species were monophyletic according to their dynamic class as determined by the virome time series, the larger tree structure shows that the dynamic class phenotype was highly paraphyletic (Figure 7). The time of year at which phage species peaked in abundance (i.e., warm vs. cool months) had larger monophyletic groups than dynamic class but was likewise paraphyletic when considering the broader structure of the tree.

### 3.6. Variance Partitioning Analysis 

**Figure 8:**
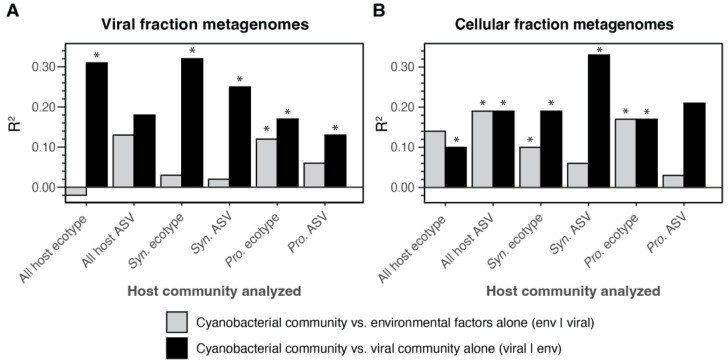
R^2^ values from variation partitioning analysis resolving the portion of host community variance explained by environmental variables or viral community structure alone in the (**A**) virome and (**B**) cellular metagenome. Analyses were performed using host community data at various taxonomic levels: all host ASVs together (*Synechococcus* and *Prochlorococcus*), all host ecotypes together, and ecotypes or ASVs within *Synechococcus* (*Syn.*) or *Prochlorococcus* (*Pro.*) separately (see *x*-axis labels). Asterisks over bars indicate significant (*p* < 0.05) R^2^ values (Table S4).

To analyze the impact of viral communities and environmental factors on host community structure, variation partitioning analysis (redundancy analysis (RDA)) and partial RDA was used [10,59]. We focused on the individual contribution of viral composition (viral|env) and environmental factors (env|viral) in explaining host community composition at the whole community level (all ASVs), ecotype level, and within ecotype level (ASVs within select ecotypes) in both *Synechococcus* and *Prochlorococcus* in separate analyses using the viral species abundances for the viromes and cellular metagenomes (Figure 8, Table S4). The viral community observed in the virome significantly explained variation for each host community analyzed at different levels of diversity (i.e., all ASVs, all ecotypes, or *Prochlorococcus* or *Synechococcus* ecotypes or ASVs). Interestingly, the viral community found in the virome explains more of the variation in *Synechococcus* host communities than in *Prochlorococcus* host communities, with environmental factors having a more profound effect on the latter (Figure 8A). When the same analysis was performed but using the viral species found in cellular metagenomes, the viral community explained more of the variation seen in host communities at the ASV level than at the ecotype level (Figure 8B).

## 4. Discussion

Here we provide a robust analysis of species-level community structure and monthly dynamics of T4-like cyanophages spanning five years at SPOT. Our analyses show that these viral communities are diverse and dominated by many low-abundance species. At the same time, we recovered several species that comprised a sizable portion, sometimes >5% of the community on average, over the course of five years, and some occasionally reached substantial levels (>10% and up to ~50%) for brief periods (Figure 4). The total cyanophage species richness in the viral metagenomes was estimated to be ~3000 and was similar to the total number of unique *gp43* sequences assembled (~2500). This suggests that diversity was well-sampled using our *gp43* assembly approach; however, we consider this a conservative estimate of actual total richness as low-abundance species may be missed in our metagenomic assembly approaches, even for these relatively high-depth metagenomes (>10 Gb per library). We also note that the *gp43* identity threshold one selects will impact the resulting number of defined species, but we have used the best available data, including relating *gp43* identity to core gene ANI, to select an appropriate threshold for delineating relevant species populations. Regardless of these limitations, our study provides a robust lower-bound estimate that T4-like cyanophage communities are composed of at least thousands of distinct species, highlighting that their phage–host interaction networks are highly diverse and complex.

Consistent with past work [11,23], we identify a significant seasonal pattern in the entire T4-like cyanophage community (Figure 1). The results here importantly differ from these prior studies by providing a specific and focused analysis of species-level community dynamics of T4-like cyanophage, whereas Ignacio-Espinoza et al. 2020 [11] focused on strain-level dynamics and tRFLP data from Chow and Fuhrman 2012 [23] (used in Ahlgren et al. 2019 [10]), could not specifically resolve cyanophage taxa. Our analysis reveals that the seasonal trend of the entire T4-like cyanophage community appears to be driven by a subset of its members, while many other phage species had various types of non-seasonal dynamics. The seasonal phages overwhelmingly belong to a cluster of ‘cool weather’ phages that peak in abundance in the late fall and winter that are correlated with *Prochlorococcus* ASVs and a ‘warm weather’ cluster of phages that peak in abundance in spring and early summer that are correlated with *Synechococcus* ASVs (Figure 6). This suggests that seasonal phages are specific to seasonal hosts.

Roughly half of the cyanophage species were not seasonal and were categorized as persistently, sporadically, or occasionally abundant. We suggest that sporadic and occasional species types have rather narrow host ranges such that they peak in abundance following a rise in the abundance of their respective particular hosts. The high abundance of persistent phages in the virome (~40% of the community on average) suggests at least two possible life histories. First, these phages may have broad and potentially cross-genera host ranges. Second, because the host community is dominated by *Synechococcus* ecotype IV and *Prochlorococcus* HLI, persistent phages may be those that specialize in infecting these dominant taxa. Increased resistance to decay may also be an important component of how and why these phages are persistent (see below). The T4-like cyanomyoviral community thus appears to be composed of species with varied life strategies that we suggest are primarily driven by differing host-range strategies, largely consistent with previous work indicating nested patterns of phage–host interactions [62,63]. Furthermore, several species from all identified categories contribute substantially (>10% and up to ~50% maximum relative abundance) to the community for only brief periods, highlighting how highly dynamic these communities are (Figure 4E,F). 

Prior analysis of the whole phage community with these same virome samples using assembled contigs identified viral contig populations (at ~98% nucleotide identity) that were detected in nearly all samples [11]. That work, however, did not conduct a detailed species-level analysis of cyanophage populations nor categorization of them as seasonal, persistent, etc., as was performed here. Within those frequently detected and relatively stable contig populations (including those of cyanophage), single nucleotide polymorphism (SNP) analysis revealed a constant overturn of variants, potentially representing Red Queen (RQH) dynamics due to continual phage–host co-evolution. This marked difference in dynamics (relatively stable vs. constant overturn) between broader and finer scales of diversity for both phages and hosts (i.e., ecotypes or species vs. variants) parallels patterns seen in our prior analysis of host cyanobacterial populations at SPOT [10] as well as a model suggested by Rodriguez-Brito et al. 2010 [8] where populations are more stable at higher taxonomic levels but cycle rapidly at finer taxonomic scales. This model, however, may require some revision, with our results here showing that cyanophage species have more complex, variable dynamics likely driven by host availability and differing host-range strategies. The previous SNP analysis of only persistent, longer contig populations in Ignacio-Espinoza et al. 2020 [11] leaves unanswered questions about how strain-level dynamics operate within the seasonal, occasional, and sporadic phages.

Phylogenetic analysis shows that the dynamic phenotypes in the viromes (seasonal, persistent, sporadic, occasional) are monophyletic for closely related species, but these traits are paraphyletic at broader phylogenetic scales. The time of year at which seasonal species peak (warm or cool months) likewise shows the same phylogenetic patterns. Under the paradigm that host range is a primary driver of viral dynamics, the fact that even relatively closely related species have different dynamics is consistent with the fact that minor changes in the genome, sometimes single nucleotide changes, can alter host range [9,64]. Complex pleiotropic effects and combinations of mutations across multiple genes that determine host range likewise may help explain how similar dynamic phenotypes may have arisen multiple times in the evolution of T4-like cyanophage populations [64]. Dekel-Bird et al. (2013) [65] saw similar phenotype-phylogenetic patterns, albeit for a different group of phage, cyanopodoviruses, and with a different phenotype. Closely related podoviruses often were monophyletic for the ecotype of the host on which they were isolated, but this pattern did not hold at broader phylogenetic clades, suggesting that host range is connected to phylogenetic relatedness of phages. It is worth noting, however, that our analysis of phenotype and phylogenetic patterns is based on a much deeper sampling of phage community diversity and with molecular methods free of potential culture-based biases.

The balance of viral species according to dynamic type differed significantly in the cellular and viral size fraction metagenomes, indicating that these samples may be capturing different processes in the life cycle of the phages. Viral metagenomes were dominated by seasonal types, while cellular metagenomes were dominated by sporadic types (Figure 3 and Figure 4). In addition, phage species were detected less frequently and at higher average community contribution in cellular metagenomes than phages in the viromes (Figure 3 and Figure 4). Together this is consistent with the cellular fraction being enriched in phages involved in an active infection. Through the lens of the KtW model, phages in the cellular metagenome may represent the “winners” at the time of sampling, whereas phages found in the virome represent a catalog of previously successful phages. Sizable overlap of phages found in the cellular metagenome and viral metagenome (Table S1) and strong correlations of species abundances between these size fractions (Figure S5) from the same sample data support that the viral metagenome contains phages very recently released by lysis. The fact that seasonal phages become undetectable for months at a time before reemerging and the sudden and ephemeral appearance of sporadic phages in the virome supports the Bank model in that many species of phages are present below our limit of detection and only rise in abundance when their preferred host is present. We suggest that these data support that both the KtW and Bank models are at play in this environment—after a successful infection cycle, phages may fall to low abundance and persist as virions until susceptible hosts are present again. Because myoviruses are generally not lysogenic and there is no evidence that cyanomyoviruses utilize this life strategy, we consider phages found in the cellular metagenome to only be involved in the lytic cycle. We also expect that the KtW and Bank models of phage–host interactions work in conjunction with RQH dynamics in complex ways, potentially operating at different levels of diversity, as noted above.

It is important to recognize that the viral metagenome likely contains a complex collection of recently released phages and those that have persisted over a range of time. As noted above, it is possible that persistent phages are observed as such in part because they are particularly resistant to degradation. Phage decay rates vary with season and location, owing to exposure in particular to damaging UV light [66,67,68,69], but how decay rates vary between cyanophage isolates or populations is not well studied. There are few studies of decay rates among marine phage isolates [67]. However, a recent study found that strains within two major lineages of cyanopodoviruses (clade A and B) did not differ significantly in their decay rates, despite these clades having clear differences in host range and lifestyle strategies [70]. Because phage decay is an important factor in the dynamics of community composition, more analysis of decay rate variation within a phylogenetic framework, especially for cyanomyoviruses, is warranted.

Consistent with viral metagenomes being enriched in previously successful phages, variation partitioning shows that the viral community in viral metagenomes explains more of the variation in host community structure across all ecotypes than the viral community captured in the cellular metagenome. However, the fact that viruses in both the virome and cellular metagenomes explained at least a portion of the variation in host community structure at both the ecotype and ASV level and with differences between *Prochlorococcus* and *Synechococcus* indicates that virus, host, and environment interactions are complex. Indeed large portions of host community variation were still unexplained by either viral or environmental factors (Table S4). There are clearly multiple additional factors not measured here that contribute to this unexplained variation, such as organic nutrients (bottom-up controls) [71,72,73], interactions with cellular predators (i.e., grazers) (top-down controls) [74,75,76], allopathic interactions between cyanobacteria (‘lateral’ controls) [77], and predation by non-T4 cyanophages such as T7 cyanophages [65]. Indeed S-TIM5-like cyanophages, a lineage of non-T4 cyanomyoviruses, exhibit variation over time [78]. However, there are fewer genomes available for non-T4 cyanophages, which makes it difficult to determine relevant ANI levels (and corresponding marker gene divergence) at which species-like populations are delineated, and this is, in part, why we have focused on T4-like cyanophage populations here.

## 5. Conclusions

In summary, this study provides a comprehensive view of phage species-level community composition and dynamics. To our knowledge, this is the first such quantitative analysis of marine T4-like cyanophage population structure and dynamics that utilizes a robust, genome-informed grouping of phage into species-like units. Our results show a diverse cyanophage community with two major groups of seasonal species that are broadly associated with *Prochlorococcus* or *Synechococcus* host ASVs. We show that while the cyanophage community as whole changes seasonally, individual cyanophage species have diverse phenotypes in terms of dynamics, and these phenotypes probably reflect that cyanophages possess an underlying diversity of host-range strategies. Phylogenetic analysis likewise indicates that these dynamic phenotype classes have emerged multiple times, likely reflecting a range of evolutionary pathways that determine host range and these phenotypes. By analyzing both cellular and viral cell fractions, we provide unique snapshots of two distinct points in the phage life cycle, those in active infection and those currently filling the bank of virions available for future infection cycles.

## Figures and Tables

**Table 1:** Number of significant host–virus and virus–virus relationships identified by eLSA time series analysis.

Category	Number of Connections
Host Ecotype—Viruses in Cellular Metagenomes	38
Host Ecotype—Viruses in Viral Fraction Metagenomes	11
Host ASV—Viruses in Cellular Metagenomes	223
Host ASV—Viruses in Viral Fraction Metagenomes	58
Host ASV—Host ASV	1524
Viruses in Viral Fraction Metagenomes—Viruses in Viral Fraction Metagenomes	905
Viruses in Viral Fraction Metagenomes—Viruses in Cellular Metagenomes	844
Viruses in Cellular Metagenomes—Viruses in Cellular Metagenomes	936

## Data Availability

Viral metagenomes are available under JGI Biosample IDs Gb0149069-Gb0149020. Sequence data for the cellular fraction metagenomes generated in this study are available at the National Center for Biotechnology Information (NCBI) under BioProject PRJNA814250 with read data submitted under Sequence Read Archive (SRA) accessions SRR18278848-SRR18278886. T4-like cyanophage *gp43* sequences assembled from metagenomes using Xander are provided in the Supplementary Materials.

## Supplementary Materials

The following supporting information can be downloaded at: https://www.mdpi.com/article/10.3390/v15020581/s1. Table S1: Sampling date and metagenome information; Table S2: Accession numbers of cyanophage genome and *gp43* sequences used in this study; Table S3: Dynamic class categorization of each species. Table S4: R2 values from variation partitioning analysis. Figure S1: Comparison of *gp43* sequence identity to core gene ANI. Figure S2: Rarefaction curves of *gp43* species. Figure S3: Average relative abundance of cumulative T4-like cyanophage species over time. Figure S4: The number of species categorized to each dynamic class, including downsampling of read coverage; Figure S5: Scatter plots of RPKM, relative abundance, and mean and maximum relative abundance in viral vs. cellular fraction samples. File S1: File containing the T4-like cyanophage *gp43* sequences assembled from metagenomes using Xander in fasta format.
