# Supplementary material for: Diverse Marine T4-like Cyanophage Communities Are Primarily Comprised of Low-Abundance Species Including Species with Distinct Seasonal, Persistent, Occasional, or Sporadic Dynamics
Source: Viruses. 2023 Feb 20;15(2):581. doi: 10.3390/v15020581 (PMC9960396; doi:10.3390/v15020581)
Supplement: Supplementary file 1 [file viruses-15-00581-s001.zip › Dart_SPOT_Viruses_supp_material/Dart_SPOT_Viruses_Supp_Material_R1.pdf]

Supplementary Material for Dart et al. "Diverse marine T4-like cyanophage communities are primarily comprised of low-abundance species with distinct seasonal, persistent, occasional or sporadic dynamics"

File S1. File, in fasta format, containing the T4-like cyanophage *gp43* sequences assembled from metagenomes using Xander.

Table S1. Sample information including date, library size, *gp43* depth, and the number of viral species.

				**Viral fraction metagenomes**				**Cellular fraction metagenomes**				
**Sample Date**	**ITS ASV data available**	**Total number of reads in library**	**No. of viral species shared between size fractions**	**Percent of reads that mapped to *gp43***	**Total *gp43* RPKM**	**No. of viral species**	**% of species also found in the cellular sample**	**Total no. of reads in library**	**Percent of reads that mapped to *gp43***	**Total *gp43* RPKM**	**No. of viral species**	**% of species also found in viral fraction sample**
May 2009	Yes	NA	NA	NA	NA	NA	NA	22996916	0.00065	1421909	37	NA
Jun 2009	Yes	NA	NA	NA	NA	NA	NA	26900748	0.00042	1660784	34	NA
Jul 2009	No	116113800	79	0.0049	2495556	182	46	26108150	0.00211	1597399	83	95
Aug 2009	No	136404488	49	0.0022	2533093	162	37	28306430	0.00080	1833675	60	82
Sep 2009	No	145676882	36	0.0063	2504016	143	28	38964758	0.00081	2032230	40	90
Oct 2009	No	148070378	NA	0.0057	2492199	180	NA	NA	NA	NA	NA	NA
Nov 2009	No	NA	NA	NA	NA	NA	NA	NA	NA	NA	NA	NA
Dec 2009	Yes	142425786	75	0.0069	2487968	196	42	30276798	0.00143	1297380	82	91
Jan 2010	Yes	NA	NA	NA	NA	NA	NA	31403772	0.00056	1517868	35	NA
Feb 2010	Yes	139674392	53	0.0043	2504793	166	36	32357404	0.00086	1227464	60	88
Mar 2010	Yes	126935932	24	0.0043	2490523	154	16	47406330	0.00030	1606528	24	100
Apr 2010	Yes	107296704	31	0.0019	2500870	97	49	44924100	0.00039	1704276	48	65
May 2010	No	109992458	NA	0.0014	2662662	62	NA	NA	NA	NA	NA	NA
Jun 2010	No	134357962	43	0.0047	2497537	165	27	28012512	0.00069	1715258	44	98
Jul 2010	Yes	157388460	52	0.0044	2455455	152	36	19321836	0.00141	1780861	55	95
Aug 2010	Yes	170333992	81	0.0075	2453894	155	59	30376372	0.00226	1890456	92	88
Sep 2010	Yes	165702952	51	0.0049	2474422	162	32	13773876	0.00149	1980964	52	98
Oct 2010	Yes	147732982	NA	0.0045	2541901	126	NA	NA	NA	NA	NA	NA
Nov 2010	Yes	173384756	25	0.0036	2553286	154	21	23533836	0.00048	2032657	33	76
Dec 2010	Yes	137194150	14	0.0033	2548851	135	12	27354550	0.00024	1780099	16	88
Jan 2011	Yes	118386432	18	0.0013	2594393	60	40	49042368	0.00021	1597328	24	75
Feb 2011	No	151278558	31	0.0041	2528740	111	35	50884410	0.00029	1759264	39	79
Mar 2011	Yes	NA	NA	NA	NA	NA	NA	10387850	0.00029	1247597	8	NA
Apr 2011	Yes	133276076	100	0.0088	2430730	136	84	57293082	0.00327	1939106	114	88
May 2011	No	139042854	54	0.0083	2424072	147	38	25713414	0.00120	1647686	56	96
Jun 2011	Yes	132644724	60	0.0043	2532560	136	50	49135112	0.00070	1876349	68	88

Jul 2011	No	134825504	88	0.0060	2465323	156	64	30862122	0.00308	1778676	100	88
Aug 2011	No	127223314	16	0.0038	2482495	139	14	15565636	0.00052	1851122	19	84
Sep 2011	No	118697928	33	0.0022	2562899	71	75	49141356	0.00060	1900052	53	62
Oct 2011	Yes	131028066	NA	0.0029	2554200	87	NA	NA	NA	NA	NA	NA
Nov 2011	No	150134260	39	0.0076	2513483	161	29	19575554	0.00090	1579206	47	83
Dec 2011	Yes	171587856	NA	0.0076	2508539	180	NA	NA	NA	NA	NA	NA
Jan 2012	Yes	139282760	87	0.0055	2478135	153	71	52809186	0.00188	1791763	108	81
Feb 2012	Yes	140166006	88	0.0066	2468609	182	54	49075018	0.00180	1798970	99	89
Mar 2012	Yes	NA	NA	NA	NA	NA	NA	37046584	0.00131	2019903	66	NA
Apr 2012	No	NA	NA	NA	NA	NA	NA	NA	NA	NA	NA	NA
May 2012	Yes	136156396	NA	0.0050	2531398	163	NA	NA	NA	NA	NA	NA
Jun 2012	Yes	163606112	109	0.0088	2447780	186	65	48477416	0.00236	1809541	120	91
Jul 2012	Yes	154660850	103	0.0069	2500639	221	53	24682512	0.00246	1784990	118	87
Aug 2012	Yes	160152640	36	0.0155	2417371	163	47	36264888	0.00101	1742575	76	47
Sep 2012	Yes	515444920	NA	0.0075	2513683	201	NA	NA	NA	NA	NA	NA
Oct 2012	Yes	NA	NA	NA	NA	NA	NA	151191326	0.00158	1377198	157	NA
Nov 2012	Yes	160169864	154	0.0104	2484180	184	102	120863368	0.00328	1467804	188	82
Dec 2012	Yes	131348436	114	0.0095	2468132	162	90	62401006	0.00222	1620876	145	79
Jan 2013	Yes	133017464	37	0.0030	2533561	109	47	34720408	0.00068	1594313	51	73
Feb 2013	Yes	134295608	56	0.0086	2444807	149	44	23218858	0.00169	1867558	65	86
Mar 2013	Yes	133767562	66	0.0037	2472945	123	63	53685386	0.00087	1735602	78	85
Apr 2013	Yes	129689028	88	0.0077	2426908	133	74	38636842	0.00305	1834998	99	89
May 2013	Yes	149622916	NA	0.0049	2497764	149	NA	NA	NA	NA	NA	NA
Jun 2013	No	112206560	NA	0.0052	2535350	141	NA	NA	NA	NA	NA	NA
Jul 2013	Yes	92909168	NA	0.0029	2504018	137	NA	NA	NA	NA	NA	NA
Aug 2013	Yes	161873630	NA	0.0045	2486676	198	NA	NA	NA	NA	NA	NA
Sep 2013	Yes	176399532	NA	0.0038	2511056	207	NA	NA	NA	NA	NA	NA
Oct 2013	Yes	NA	NA	NA	NA	NA	NA	NA	NA	NA	NA	NA
Nov 2013	Yes	135269650	NA	0.0057	2500560	195	NA	NA	NA	NA	NA	NA
Dec 2013	Yes	147785986	NA	0.0058	2498068	135	NA	NA	NA	NA	NA	NA
Jan 2014	No	153897306	NA	0.0087	2496108	184	NA	NA	NA	NA	NA	NA
Feb 2014	No	162325522	NA	0.0082	2475969	170	NA	NA	NA	NA	NA	NA
Mar 2014	No	NA	NA	NA	NA	NA	NA	NA	NA	NA	NA	NA
Apr 2014	No	175307902	NA	0.0010	2494438	120	NA	NA	NA	NA	NA	NA
May 2014	No	78821612	NA	0.0036	2463488	141	NA	NA	NA	NA	NA	NA
Jun 2014	No	NA	NA	NA	NA	NA	NA	NA	NA	NA	NA	NA
Jul 2014	No	NA	NA	NA	NA	NA	NA	NA	NA	NA	NA	NA
Aug 2014	No	NA	NA	NA	NA	NA	NA	NA	NA	NA	NA	NA
Sep 2014	No	107737506	NA	0.0111	2465536	165	NA	NA	NA	NA	NA	NA

Table S2. Accession numbers of cyanophage genome and *gp43* sequences used in study.

**Cyanophage genomes used in this study**
AJ630128.1,AY939844.2,AY940168.2,DQ149023.2,FM207411.1,GU071092.1,GU071094.1,GU071095.1,GU071096.1,GU071097.1,GU071098.1,GU071099.1,GU071101.1,GU071103.1,GU071105.1,GU071106.1,GU071108.1,GU075905.1,HQ316583.1,HQ316603.1,HQ317290.1,HQ317291.1,HQ317292.1,HQ317383.1,HQ317385.1,HQ317391.1,HQ337021.1,HQ615693.1,HQ632825.1,HQ633071.1,HQ634174.1,HQ634175.1,HQ634176.1,HQ634177.1,HQ634178.1,HQ634189.1,HQ634190.1,HQ634191.1,HQ634193.1,JF974288.1,JF974289.1,JF974292.1,JF974293.1,JN371768.1,JN371769.1,KF156338.1,KF156339.1,KF156340.1,KJ019026.1,KJ019027.1,KJ019028.1,KJ019029.1,KJ019030.1,KJ019031.1,KJ019032.1,KJ019033.1,KJ019034.1,KJ019035.1,KJ019036.1,KJ019037.1,KJ019038.1,KJ019039.1,KJ019040.1,KJ019041.1,KJ019042.1,KJ019043.1,KJ019044.1,KJ019045.1,KJ019046.1,KJ019047.1,KJ019048.1,KJ019049.1,KJ019050.1,KJ019051.1,KJ019052.1,KJ019053.1,KJ019054.1,KJ019055.1,KJ019056.1,KJ019057.1,KJ019058.1,KJ019059.1,KJ019060.1,KJ019061.1,KJ019062.1,KJ019063.1,KJ019064.1,KJ019065.1,KJ019066.1,KJ019067.1,KJ019068.1,KJ019069.1,KJ019070.1,KJ019071.1,KJ019072.1,KJ019073.1,KJ019074.1,KJ019075.1,KJ019076.1,KJ019077.1,KJ019078.1,KJ019079.1,KJ019080.1,KJ019082.1,KJ019083.1,KJ019084.1,KJ019085.1,KJ019086.1,KJ019087.1,KJ019088.1,KJ019089.1,KJ019090.1,KJ019091.1,KJ019092.1,KJ019093.1,KJ019094.1,KJ019095.1,KJ019096.1,KJ019097.1,KJ019098.1,KJ019099.1,KJ019100.1,KJ019101.1,KJ019102.1,KJ019103.1,KJ019104.1,KJ019105.1,KJ019106.1,KJ019107.1,KJ019108.1,KJ019109.1,KJ019110.1,KJ019111.1,KJ019112.1,KJ019113.1,KJ019114.1,KJ019115.1,KJ019116.1,KJ019117.1,KJ019118.1,KJ019119.1,KJ019120.1,KJ019121.1,KJ019122.1,KJ019123.1,KJ019124.1,KJ019125.1,KJ019126.1,KJ019127.1,KJ019128.1,KJ019129.1,KJ019130.1,KJ019131.1,KJ019132.1,KJ019133.1,KJ019134.1,KJ019135.1,KJ019136.1,KJ019137.1,KJ019138.1,KJ019139.1,KJ019140.1,KJ019141.1,KJ019142.1,KJ019143.1,KJ019144.1,KJ019145.1,KJ019146.1,KJ019147.1,KJ019148.1,KJ019149.1,KJ019150.1,KJ019151.1,KJ019152.1,KJ019153.1,KJ019154.1,KJ019155.1,KJ019156.1,KJ019157.1,KJ019158.1,KJ019159.1,KJ019160.1,KJ019161.1,KJ019162.1,KJ019163.1,KJ019164.1,KJ019165.1,KM359505.1,KP211958.1,KU594605.1,KU594607.1,KU686192.1,KU686193.1,KU686194.1,KU686195.1,KU686196.1,KU686197.1,KU686198.1,KU686199.1,KU686200.1,KU686201.1,KU686202.1,KU686203.1,KU686204.1,KU686205.1,KU686206.1,KU686207.1,KU686208.1,KU686209.1,KU686210.1,KU686211.1,KU686212.1,KU686213.1,KX349226.1,KX349227.1,KX349228.1,KX349229.1,KX349230.1,KX349231.1,KX349232.1,KX349233.1,KX349234.1,KX349235.1,KX349236.1,KX349237.1,KX349238.1,KX349239.1,KX349240.1,KX349241.1,KX349242.1,KX349243.1,KX349244.1,KX349245.1,KX349246.1,KX349247.1,KX349248.1,KX349249.1,KX349250.1,KX349251.1,KX349252.1,KX349253.1,KX349254.1,KX349255.1,KX349256.1,KX349257.1,KX349258.1,KX349259.1,KX349260.1,KX349261.1,KX349262.1,KX349263.1,KX349264.1,KX349265.1,KX349266.1,KX349267.1,KX349268.1,KX349269.1,KX349270.1,KX349271.1,KX349272.1,KX349273.1,KX349274.1,KX349275.1,KX349276.1,KX349277.1,KX349278.1,KX349279.1,KX349280.1,KX349281.1,KX349282.1,KX349283.1,KX349284.1,KX349285.1,KX349286.1,KX349287.1,KX349288.1,KX349289.1,KX349290.1,KX349291.1,KX349292.1,KX349293.1,KX349294.1,KX349295.1,KX349296.1,KX349297.1,KX349298.1,KX349299.1,KX349300.1,KX349301.1,KX349302.1,KX349303.1,KX349304.1,KX349305.1,KX349306.1,KX349307.1,KX349308.1,KX349309.1,KX349310.1,KX349311.1,KX349312.1,KX349313.1,KX349314.1,KX349315.1,KX349316.1,KX349317.1,KX349318.1,KX349319.1,KX349320.1,KX349321.1,KX349322.1,KX349323.1,KX349324.1,KX349325.1,KX349326.1,KX349327.1,KY945241.1,LN828717.1,MF351863.1
**Cyanophage *gp43* gene sequence amplicons from environmental samples used in this study**
FJ874707.1,FJ874706.1,FJ874705.1,FJ874704.1,FJ874703.1,FJ874696.1,FJ874697.1,FJ874698.1,FJ874699.1,FJ874700.1,FJ874701.1,FJ874702.1,FJ874684.1,FJ874685.1,FJ874686.1,FJ874687.1,FJ874688.1,FJ874689.1,FJ874690.1,FJ874691.1,FJ874692.1,FJ874693.1,FJ874694.1,FJ874695.1,FJ874732.1,FJ874733.1,FJ874674.1,FJ874675.1,FJ874676.1,FJ874677.1,FJ874678.1,FJ874679.1,FJ874680.1,FJ874681.1,FJ874682.1,FJ874683.1,FJ874717.1,FJ874718.1,FJ874719.1,FJ874720.1,FJ874721.1,FJ874722.1,FJ874723.1,FJ874724.1,FJ874725.1,FJ874726.1,FJ874727.1,FJ874728.1,FJ874729.1,FJ874730.1,FJ874731.1,FJ874716.1,FJ874715.1,JX911892.1,JX911893.1,JX911894.1,FJ874708.1,FJ874709.1,FJ874710.1,FJ874711.1,FJ874712.1,FJ874713.1,FJ874714.1,JX911883.1,JX911884.1,JX911885.1,JX911886.1,JX911887.1,JX911888.1,JX911889.1,JX911890.1,JX911891.1,JX911868.1,JX911869.1,JX911870.1,JX911871.1,JX911872.1,JX911873.1,JX911874.1,JX911875.1,JX911876.1,JX911877.1,JX911878.1,JX911879.1,JX911880.1,JX911881.1,JX911882.1,JX911853.1,JX911854.1,JX911855.1,JX911856.1,JX911857.1,JX911858.1,JX911859.1,JX911861.1,JX911860.1,JX911862.1,JX911863.1,JX911864.1,JX911865.1,JX911866.1,JX911867.1,JX911837.1,JX911838.1,JX911839.1,JX911840.1,JX911841.1,JX911842.1,JX911843.1,JX911844.1,JX911845.1,JX911846.1,JX911847.1,JX911848.1,JX911849.1,JX911850.1,JX911851.1,JX911852.1,JX911824.1,JX911825.1,JX911826.1,JX911827.1,JX911828.1,JX911829.1,JX911830.1,JX911831.1,JX911832.1,JX911834.1,JX911833.1,JX911835.1,JX911836.1,JX911810.1,JX911811.1,JX911812.1,JX911813.1,JX911814.1,JX911815.1,JX911816.1,JX911823.1,JX911822.1,JX911821.1,JX911820.1,JX911819.1,JX911818.1,JX911817.1,JX911795.1,JX911796.1,JX911797.1,JX911798.1,JX911799.1,JX911800.1,JX911801.1,JX911802.1,JX911803.1,JX911804.1,JX911805.1,JX911806.1,JX911807.1,JX911808.1,JX911809.1,JX911780.1,JX911781.1,JX911782.1,JX911783.1,JX911784.1,JX911785.1,JX911786.1,JX911787.1,JX911788.1,JX911789.1,JX911794.1,JX911793.1,JX911792.1,JX911791.1,JX911790.1,JX911766.1,JX911767.1,JX911768.1,JX911769.1,JX911770.1,JX911771.1,JX911772.1,JX911773.1,JX911774.1,JX911775.1,JX911776.1,JX911777.1,JX911778.1,JX911779.1,JX911755.1,JX911756.1,JX911757.1,JX911758.1,JX911759.1,JX911760.1,JX911761.1,JX911763.1,JX911762.1,JX911764.1,JX911765.1

Table S3. The dynamic class categorization of each viral species according to the virome or cellular metagenome. Highlighted species are those that fit the persistent criterion and have a significant seasonal spline (*n*=12).

**Phage Species**	**Virome Dynamic Category**	**Cellular Metagenome Dynamic Category**
APR2010meta_g43_contig_1756_contig_1757	occasional	occasional
APR2010meta_g43_contig_5750_contig_5751	occasional	sporadic
APR2010meta_g43_contig_686_contig_687	occasional	sporadic
APR2011meta_g43_contig_17406_contig_17407	occasional	occasional
APR2011meta_g43_contig_2976_contig_2977	occasional	occasional
APR2011meta_g43_contig_2990_contig_2991	occasional	sporadic
APR2013meta_g43_contig_2306_contig_2307	occasional	occasional
APRIL2011_g43_contig_2434_contig_2435	occasional	sporadic
APRIL2011_g43_contig_2524_contig_2525	occasional	occasional
AUG2009_g43_contig_3244_contig_3245	occasional	sporadic
AUG2009_g43_contig_6796_contig_6797	occasional	sporadic
AUG2009meta_g43_contig_6_contig_7	occasional	sporadic
AUG2009meta_g43_contig_70_contig_71	occasional	sporadic
AUG2011_g43_contig_2224_contig_2225	occasional	sporadic
AUG2012meta_g43_contig_5296_contig_5297	occasional	seasonal
AUG2012meta_g43_contig_6_contig_7	occasional	occasional
AUG2012meta_g43_contig_6308_contig_6309	occasional	seasonal
AUG2013_g43_contig_3636_contig_3637	occasional	sporadic
AUG2013_g43_contig_806_contig_807	occasional	sporadic
DEC2009_g43_contig_6362_contig_6363	occasional	occasional
DEC2011_g43_contig_27142_contig_27143	occasional	Not found in cellular metagenome
DEC2011_g43_contig_27756_contig_27757	occasional	sporadic
DEC2012meta_g43_contig_102_contig_103	occasional	sporadic
DEC2012meta_g43_contig_11250_contig_11251	occasional	sporadic
DEC2012meta_g43_contig_22134_contig_22135	occasional	occasional
DEC2012meta_g43_contig_4830_contig_4831	occasional	occasional
DEC2013_g43_contig_998_contig_999	occasional	seasonal
FEB2010_g43_contig_3428_contig_3429	occasional	sporadic
FEB2013meta_g43_contig_2200_contig_2201	occasional	occasional
Ga0181379_1000073bs	occasional	sporadic
Ga0181379_1000119bs	occasional	sporadic
Ga0181379_1024068bs	occasional	occasional
Ga0181379_1078211bs	occasional	occasional
Ga0181385_1029211bs	occasional	sporadic
Ga0181386_1126711bs	occasional	sporadic
Ga0181389_1000663bs	occasional	seasonal
Ga0181389_1105770bs	occasional	Not found in cellular metagenome
Ga0181398_1000002bs	occasional	occasional
Ga0181398_1012449bs	occasional	sporadic
Ga0181398_1035252bs	occasional	occasional
Ga0181399_1015076bs	occasional	occasional
Ga0181399_1176683bs	occasional	occasional
Ga0181400_1014569bs	occasional	sporadic
Ga0181404_1130934bs	occasional	sporadic
Ga0181406_1025137bs	occasional	occasional
Ga0181406_1054155bs	occasional	seasonal
Ga0181406_1072413bs	occasional	occasional

Ga0181407_1008799bs	occasional	occasional
Ga0181409_1027155bs	occasional	sporadic
Ga0181410_1183833bs	occasional	occasional
Ga0181411_1014521bs	occasional	seasonal
Ga0181411_1032064bs	occasional	sporadic
Ga0181411_1042452bs	occasional	occasional
Ga0181411_1262948bs	occasional	occasional
Ga0181412_1007830bs	occasional	occasional
Ga0181412_1011781bs	occasional	occasional
Ga0181412_1014409bs	occasional	occasional
Ga0181412_1049517bs	occasional	sporadic
Ga0181413_1001400bs	occasional	seasonal
Ga0181414_1020179bs	occasional	occasional
Ga0181414_1105086bs	occasional	sporadic
Ga0181414_1149305bs	occasional	sporadic
Ga0181415_1011777bs	occasional	sporadic
Ga0181415_1012161bs	occasional	occasional
Ga0181415_1098829bs	occasional	sporadic
Ga0181417_1004373bs	occasional	seasonal
Ga0181418_1011334bs	occasional	occasional
Ga0181420_1011385bs	occasional	sporadic
Ga0181420_1014304bs	occasional	sporadic
Ga0181422_1120276bs	occasional	sporadic
Ga0181423_1479109bs	occasional	sporadic
Ga0181425_1005938bs	occasional	seasonal
Ga0181427_1004745bs	occasional	sporadic
Ga0181427_1181746bs	occasional	sporadic
Ga0181431_1191606bs	occasional	sporadic
Ga0187218_1099679bs	occasional	occasional
Ga0187219_1070018bs	occasional	occasional
Ga0187220_1147702bs	occasional	sporadic
Ga0187221_1046637bs	occasional	sporadic
Ga0187221_1220806bs	occasional	sporadic
JUL2012meta_g43_contig_7842_contig_7843	occasional	sporadic
JUN2012meta_g43_contig_3096_contig_3097	occasional	occasional
JUNE2013_g43_contig_1856_contig_1857	occasional	occasional
MAR2012meta_g43_contig_1802_contig_1803	occasional	occasional
MARCH2010_g43_contig_19402_contig_19403	occasional	occasional
MAY2013_g43_contig_16632_contig_16633	occasional	occasional
MAY2013_g43_contig_86_contig_87	occasional	Not found in cellular metagenome
NOV2010meta_g43_contig_1164_contig_1165	occasional	Not found in cellular metagenome
NOV2010meta_g43_contig_668_contig_669	occasional	occasional
NOV2012_g43_contig_1192_contig_1193	occasional	sporadic
NOV2012_g43_contig_16146_contig_16147	occasional	sporadic
NOV2013_g43_contig_10492_contig_10493	occasional	sporadic
OCT2010_g43_contig_2626_contig_2627	occasional	occasional
OCT2012meta_g43_contig_15748_contig_15749	occasional	sporadic
SEP2011meta_g43_contig_4244_contig_4245	occasional	occasional
SEPT2013_g43_contig_3928_contig_3929	occasional	sporadic
SEPT2014_g43_contig_4386_contig_4387	occasional	sporadic
SEPT2014_g43_contig_4626_contig_4627	occasional	sporadic
APR2010_g43_contig_1666_contig_1667	persistent	occasional
APR2010meta_g43_contig_1204_contig_1205	persistent	seasonal
APR2010meta_g43_contig_1210_contig_1211	persistent	occasional
APR2010meta_g43_contig_168_contig_169	persistent	seasonal
APR2011meta_g43_contig_16578_contig_16579	persistent	occasional

APR2011meta_g43_contig_2826_contig_2827	persistent	occasional
APR2013meta_g43_contig_10_contig_11	persistent	occasional
AUG2009_g43_contig_3196_contig_3197	persistent	persistent
AUG2009meta_g43_contig_1382_contig_1383	persistent	occasional
AUG2010_g43_contig_16066_contig_16067	persistent	occasional
AUG2011_g43_contig_760_contig_761	persistent	occasional
AUG2012_g43_contig_23330_contig_23331	persistent	sporadic
AUG2012meta_g43_contig_2074_contig_2075	persistent	occasional
DEC2009meta_g43_contig_4946_contig_4947	persistent	seasonal
DEC2009meta_g43_contig_846_contig_847	persistent	occasional
DEC2013_g43_contig_2926_contig_2927	persistent	occasional
FEB2012meta_g43_contig_3218_contig_3219	persistent	occasional
Ga0181379_1030656bs	persistent	occasional
Ga0181387_1041238bs	persistent	seasonal
Ga0181388_1004303bs	persistent	occasional
Ga0181393_1106242bs	persistent	occasional
Ga0181397_1190031bs	persistent	occasional
Ga0181400_1039600bs	persistent	seasonal
Ga0181405_1071392bs	persistent	occasional
Ga0181405_1159557bs	persistent	occasional
Ga0181415_1180415bs	persistent	seasonal
Ga0181420_1028594bs	persistent	occasional
JAN2012meta_g43_contig_3468_contig_3469	persistent	occasional
JUNE2012_g43_contig_876_contig_877	persistent	occasional
MAY2012_g43_contig_3340_contig_3341	persistent	occasional
NOV2012meta_g43_contig_45718_contig_45719	persistent	persistent
APR2010_g43_contig_1360_contig_1361	seasonal	occasional
APR2011meta_g43_contig_100_contig_101	seasonal	occasional
APR2011meta_g43_contig_15362_contig_15363	seasonal	occasional
APR2011meta_g43_contig_16040_contig_16041	seasonal	seasonal
APR2011meta_g43_contig_2814_contig_2815	seasonal	occasional
APR2011meta_g43_contig_5562_contig_5563	seasonal	occasional
APR2011meta_g43_contig_6928_contig_6929	seasonal	sporadic
APR2011meta_g43_contig_6950_contig_6951	seasonal	sporadic
APR2013meta_g43_contig_1006_contig_1007	seasonal	occasional
APR2013meta_g43_contig_11272_contig_11273	seasonal	occasional
APR2013meta_g43_contig_11976_contig_11977	seasonal	occasional
APRIL2011_g43_contig_5558_contig_5559	seasonal	sporadic
APRIL2013_g43_contig_9510_contig_9511	seasonal	sporadic
AUG2009_g43_contig_14954_contig_14955	seasonal	sporadic
AUG2009_g43_contig_2706_contig_2707	seasonal	sporadic
AUG2009_g43_contig_462_contig_463	seasonal	seasonal
AUG2009_g43_contig_66_contig_67	seasonal	sporadic
AUG2011_g43_contig_2276_contig_2277	seasonal	seasonal
AUG2012_g43_contig_126_contig_127	seasonal	sporadic
AUG2012meta_g43_contig_5304_contig_5305	seasonal	seasonal
AUG2013_g43_contig_648_contig_649	seasonal	occasional
DEC2009_g43_contig_22484_contig_22485	seasonal	occasional
DEC2009meta_g43_contig_12622_contig_12623	seasonal	sporadic
DEC2009meta_g43_contig_17638_contig_17639	seasonal	seasonal
DEC2010_g43_contig_2388_contig_2389	seasonal	sporadic
DEC2010_g43_contig_6908_contig_6909	seasonal	sporadic
DEC2010_g43_contig_72_contig_73	seasonal	seasonal
DEC2011_g43_contig_6248_contig_6249	seasonal	seasonal
DEC2012_g43_contig_18258_contig_18259	seasonal	sporadic
DEC2012_g43_contig_20320_contig_20321	seasonal	sporadic

DEC2012_g43_contig_572_contig_573	seasonal	occasional
DEC2012_g43_contig_6902_contig_6903	seasonal	sporadic
DEC2012meta_g43_contig_2120_contig_2121	seasonal	sporadic
DEC2012meta_g43_contig_4988_contig_4989	seasonal	seasonal
FEB2010_g43_contig_4670_contig_4671	seasonal	occasional
FEB2011_g43_contig_1556_contig_1557	seasonal	occasional
FEB2013meta_g43_contig_952_contig_953	seasonal	seasonal
FEB2014_g43_contig_248_contig_249	seasonal	sporadic
Ga0181379_1000218bs	seasonal	occasional
Ga0181379_1000684bs	seasonal	seasonal
Ga0181379_1002266bs	seasonal	seasonal
Ga0181379_1002463bs	seasonal	occasional
Ga0181379_1002805bs	seasonal	seasonal
Ga0181379_1004929bs	seasonal	occasional
Ga0181379_1005526bs	seasonal	seasonal
Ga0181379_1006259bs	seasonal	seasonal
Ga0181379_1006576bs	seasonal	occasional
Ga0181379_1007630bs	seasonal	sporadic
Ga0181379_1014234bs	seasonal	seasonal
Ga0181379_1014881bs	seasonal	seasonal
Ga0181379_1018033bs	seasonal	occasional
Ga0181379_1028581bs	seasonal	sporadic
Ga0181379_1030172bs	seasonal	seasonal
Ga0181379_1114691bs	seasonal	seasonal
Ga0181379_1254866bs	seasonal	sporadic
Ga0181379_1434689bs	seasonal	sporadic
Ga0181380_1147619bs	seasonal	seasonal
Ga0181385_1000001bs	seasonal	sporadic
Ga0181385_1007307bs	seasonal	occasional
Ga0181385_1015215bs	seasonal	sporadic
Ga0181385_1020752bs	seasonal	sporadic
Ga0181385_1030233bs	seasonal	occasional
Ga0181385_1038691bs	seasonal	occasional
Ga0181385_1069945bs	seasonal	seasonal
Ga0181385_1243399bs	seasonal	sporadic
Ga0181385_1324766bs	seasonal	sporadic
Ga0181386_1011570bs	seasonal	sporadic
Ga0181386_1024097bs	seasonal	occasional
Ga0181386_1070110bs	seasonal	seasonal
Ga0181387_1030261bs	seasonal	sporadic
Ga0181389_1003911bs	seasonal	sporadic
Ga0181393_1032910bs	seasonal	sporadic
Ga0181393_1165529bs	seasonal	seasonal
Ga0181394_1015448bs	seasonal	seasonal
Ga0181394_1042751bs	seasonal	seasonal
Ga0181394_1048085bs	seasonal	sporadic
Ga0181395_1059861bs	seasonal	seasonal
Ga0181395_1130955bs	seasonal	sporadic
Ga0181397_1049832bs	seasonal	occasional
Ga0181397_1055744bs	seasonal	seasonal
Ga0181397_1090099bs	seasonal	occasional
Ga0181398_1003828bs	seasonal	occasional
Ga0181398_1003939bs	seasonal	occasional
Ga0181398_1005838bs	seasonal	occasional
Ga0181398_1007555bs	seasonal	seasonal
Ga0181398_1033708bs	seasonal	seasonal

Ga0181398_1085201bs	seasonal	occasional
Ga0181399_1002291bs	seasonal	seasonal
Ga0181399_1017051bs	seasonal	occasional
Ga0181399_1040767bs	seasonal	seasonal
Ga0181399_1054426bs	seasonal	seasonal
Ga0181399_1173509bs	seasonal	seasonal
Ga0181399_1263175bs	seasonal	occasional
Ga0181400_1003441bs	seasonal	sporadic
Ga0181400_1014965bs	seasonal	seasonal
Ga0181400_1044096bs	seasonal	sporadic
Ga0181400_1085153bs	seasonal	occasional
Ga0181400_1199437bs	seasonal	occasional
Ga0181401_1000353bs	seasonal	occasional
Ga0181401_1061776bs	seasonal	occasional
Ga0181402_1014322bs	seasonal	occasional
Ga0181402_1209020bs	seasonal	seasonal
Ga0181405_1002784bs	seasonal	occasional
Ga0181405_1007446bs	seasonal	sporadic
Ga0181405_1023051bs	seasonal	sporadic
Ga0181406_1014781bs	seasonal	seasonal
Ga0181406_1017526bs	seasonal	sporadic
Ga0181406_1055040bs	seasonal	seasonal
Ga0181406_1213993bs	seasonal	occasional
Ga0181408_1007423bs	seasonal	occasional
Ga0181408_1019757bs	seasonal	seasonal
Ga0181408_1063904bs	seasonal	sporadic
Ga0181408_1124031bs	seasonal	sporadic
Ga0181409_1055944bs	seasonal	sporadic
Ga0181409_1076540bs	seasonal	seasonal
Ga0181409_1083566bs	seasonal	sporadic
Ga0181410_1000492bs	seasonal	sporadic
Ga0181410_1010765bs	seasonal	seasonal
Ga0181410_1016659bs	seasonal	seasonal
Ga0181410_1123612bs	seasonal	sporadic
Ga0181411_1008102bs	seasonal	sporadic
Ga0181411_1014315bs	seasonal	seasonal
Ga0181411_1036307bs	seasonal	seasonal
Ga0181411_1041542bs	seasonal	seasonal
Ga0181411_1100704bs	seasonal	occasional
Ga0181412_1000360bs	seasonal	sporadic
Ga0181412_1020511bs	seasonal	occasional
Ga0181413_1001476bs	seasonal	sporadic
Ga0181413_1014952bs	seasonal	occasional
Ga0181413_1015007bs	seasonal	sporadic
Ga0181413_1021728bs	seasonal	sporadic
Ga0181413_1062817bs	seasonal	sporadic
Ga0181413_1129136bs	seasonal	occasional
Ga0181413_1208977bs	seasonal	occasional
Ga0181414_1014471bs	seasonal	occasional
Ga0181414_1014950bs	seasonal	sporadic
Ga0181414_1020475bs	seasonal	sporadic
Ga0181414_1100124bs	seasonal	occasional
Ga0181414_1111542bs	seasonal	sporadic
Ga0181415_1000036bs	seasonal	occasional
Ga0181415_1007876bs	seasonal	sporadic
Ga0181419_1006487bs	seasonal	sporadic

Ga0181419_1066017bs	seasonal	occasional
Ga0181420_1027863bs	seasonal	occasional
Ga0181420_1076274bs	seasonal	occasional
Ga0181421_1069880bs	seasonal	seasonal
Ga0181421_1150983bs	seasonal	seasonal
Ga0181422_1020236bs	seasonal	sporadic
Ga0181422_1056299bs	seasonal	occasional
Ga0181424_10011512bs	seasonal	seasonal
Ga0181425_1000096bs	seasonal	sporadic
Ga0181425_1022215bs	seasonal	sporadic
Ga0181426_1006399bs	seasonal	sporadic
Ga0181426_1026699bs	seasonal	sporadic
Ga0181427_1039755bs	seasonal	occasional
Ga0181427_1219897bs	seasonal	sporadic
Ga0181428_1035284bs	seasonal	sporadic
Ga0181428_1045080bs	seasonal	sporadic
Ga0181428_1061155bs	seasonal	sporadic
Ga0181433_1038612bs	seasonal	sporadic
Ga0181433_1051187bs	seasonal	sporadic
Ga0187219_1003570bs	seasonal	sporadic
Ga0187220_1016318bs	seasonal	occasional
Ga0187220_1201201bs	seasonal	sporadic
Ga0187221_1019522bs	seasonal	sporadic
Ga0187221_1036632bs	seasonal	sporadic
Ga0187221_1133050bs	seasonal	seasonal
JAN2010meta_g43_contig_6_contig_7	seasonal	sporadic
JULY2013_g43_contig_1798_contig_1799	seasonal	occasional
JUNE2011_g43_contig_14284_contig_14285	seasonal	sporadic
JUNE2013_g43_contig_1892_contig_1893	seasonal	occasional
JUNE2013_g43_contig_1902_contig_1903	seasonal	occasional
KJ019085.1bs	seasonal	sporadic
MAY2012_g43_contig_20_contig_21	seasonal	sporadic
NOV2011meta_g43_contig_6318_contig_6319	seasonal	sporadic
NOV2013_g43_contig_222_contig_223	seasonal	sporadic
SEPT2014_g43_contig_810_contig_811	seasonal	sporadic
APRIL2013_g43_contig_7638_contig_7639	stochastic	Not found in cellular metagenome
AUG2009_g43_contig_2438_contig_2439	stochastic	sporadic
AUG2012meta_g43_contig_112_contig_113	stochastic	sporadic
AUG2013_g43_contig_13876_contig_13877	stochastic	sporadic
DEC2009_g43_contig_20_contig_21	stochastic	sporadic
DEC2011_g43_contig_8184_contig_8185	stochastic	sporadic
DEC2012_g43_contig_4042_contig_4043	stochastic	sporadic
DEC2012meta_g43_contig_7668_contig_7669	stochastic	sporadic
DEC2013_g43_contig_50_contig_51	stochastic	Not found in cellular metagenome
FEB2010meta_g43_contig_7140_contig_7141	stochastic	sporadic
FEB2010meta_g43_contig_7396_contig_7397	stochastic	sporadic
FEB2012_g43_contig_4036_contig_4037	stochastic	sporadic
FEB2012meta_g43_contig_124_contig_125	stochastic	sporadic
FEB2013meta_g43_contig_4410_contig_4411	stochastic	sporadic
Ga0181379_1000597bs	stochastic	sporadic
Ga0181379_1002501bs	stochastic	sporadic
Ga0181379_1002687bs	stochastic	sporadic
Ga0181379_1003739bs	stochastic	sporadic
Ga0181379_1005592bs	stochastic	sporadic
Ga0181379_1019331bs	stochastic	sporadic
Ga0181379_1034898bs	stochastic	sporadic

Ga0181379_1042486bs	stochastic	sporadic
Ga0181389_1076305bs	stochastic	sporadic
Ga0181394_1044996bs	stochastic	sporadic
Ga0181397_1015530bs	stochastic	sporadic
Ga0181402_1005406bs	stochastic	sporadic
Ga0181406_1056874bs	stochastic	sporadic
Ga0181414_1006279bs	stochastic	sporadic
Ga0181414_1011143bs	stochastic	sporadic
Ga0181414_1026333bs	stochastic	sporadic
Ga0181415_1001468bs	stochastic	occasional
Ga0181428_1114155bs	stochastic	sporadic
Ga0181433_1013728bs	stochastic	sporadic
Ga0181433_1066998bs	stochastic	Not found in cellular metagenome
Ga0187217_1063577bs	stochastic	sporadic
Ga0187219_1000539bs	stochastic	sporadic
Ga0187220_1067493bs	stochastic	sporadic
JAN2013meta_g43_contig_42_contig_43	stochastic	sporadic
JUL2009meta_g43_contig_126_contig_127	stochastic	sporadic
JUL2012meta_g43_contig_10380_contig_10381	stochastic	sporadic
JULY2010_g43_contig_1590_contig_1591	stochastic	sporadic
JUN2012meta_g43_contig_182_contig_183	stochastic	sporadic
MAY2013_g43_contig_7148_contig_7149	stochastic	occasional
NOV2011meta_g43_contig_2130_contig_2131	stochastic	sporadic
NOV2011meta_g43_contig_28_contig_29	stochastic	sporadic
OCT2012meta_g43_contig_1210_contig_1211	stochastic	sporadic
OCT2012meta_g43_contig_2938_contig_2939	stochastic	sporadic
OCT2012meta_g43_contig_86_contig_87	stochastic	sporadic

Table S4. R^2 values from variation partitioning analysis resolving the portion of host community variance explained by environmental variables and viral community structure in the virome and cellular metagenomes.

	RDA		Partial RDA		
	Cyanobacteria community vs. environmental factors	Cyanobacteria community vs. viral community	Cyanobacterial vs. environmental factors alone (env|virome)	Cyanobacterial vs. viral factors alone (virome|env)	Unexplained Variance
**Host community level tested with viral community from virome**					
All host ecotype	0.34***	0.66***	-0.02	0.31*	0.35
All host ASV	0.48***	0.54***	0.13	0.18	0.34
Syn. ecotype	0.33**	0.62***	0.03	0.32*	0.35
Syn. ASV	0.23***	0.46***	0.02	0.25**	0.52
Pro. Ecotype	0.73***	0.78**	0.12*	0.17*	0.10
Pro. ASV	0.37***	0.44***	0.06	0.13*	0.50
**Host community level tested with viral community from cellular metagenome**					
All host ecotype	0.34***	0.30***	0.14	0.10*	0.56
All host ASV	0.50***	0.51***	0.19*	0.19**	0.3
Syn. ecotype	0.33**	0.42***	0.10*	0.19**	0.48
Syn. ASV	0.23***	0.50***	0.06	0.33***	0.44
Pro. Ecotype	0.70***	0.71***	0.17**	0.17**	0.12
Pro. ASV	0.37***	0.54***	0.03	0.21	0.43

Asterisks indicate the level of ANOVA support for the estimated contribution to host variance: $*p < 0.05$; $**p < 0.01$; $***p \leq 0.001$

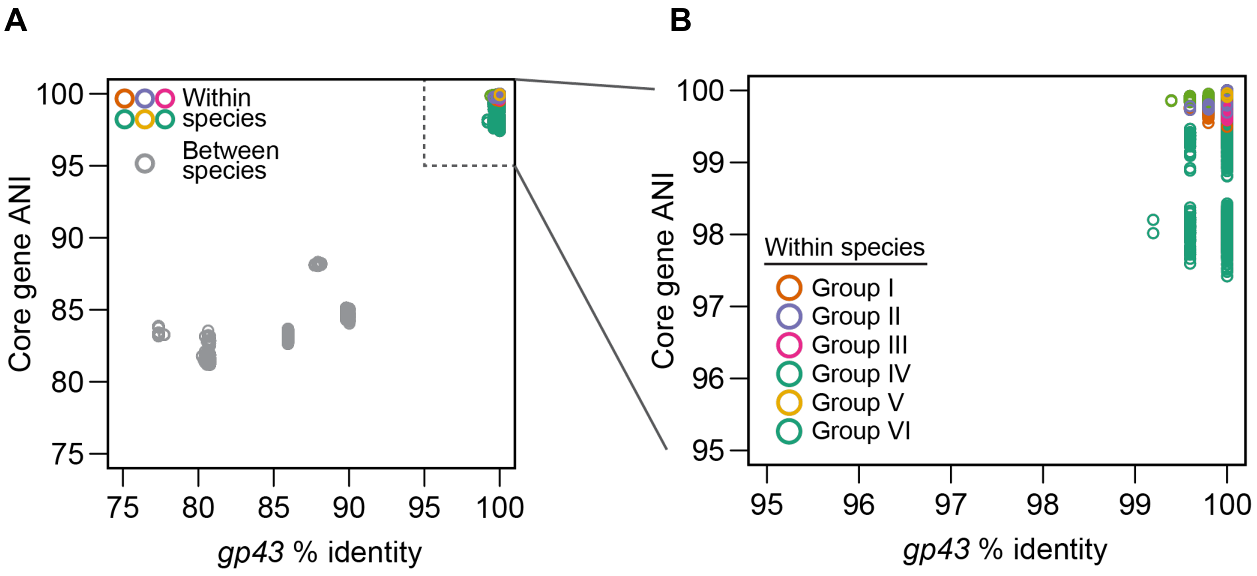

Figure S1. Comparison of *gp43* percent identity vs. core gene average nucleotide identity (ANI) among 142 complete T4-like cyanophage genomes from Gregory *et al.* 2016. Each point represents a pairwise comparison between two cyanophage strains where points are color coded according to interspecies (grey) or intraspecies (colors) comparisons. The six populations in this dataset referred to as Groups I-VI in Gregory *et al.* 2016 and represent species populations using the 95% ANI threshold in Deng et al. 2014. Panel **B** depicts a closer view of **A** (dashed box) at 95-100% *gp43* identity and ANI. *gp43* % identity was determined with blastn for the region amplified by the *gp43* primers gp43For and gp43Rev (see Methods in main text). ANI values were computed using 51 core genes as determined and analyzed in Gregory *et al.* 2016, requiring >60% alignment and >40% percent identity to be included in the average.

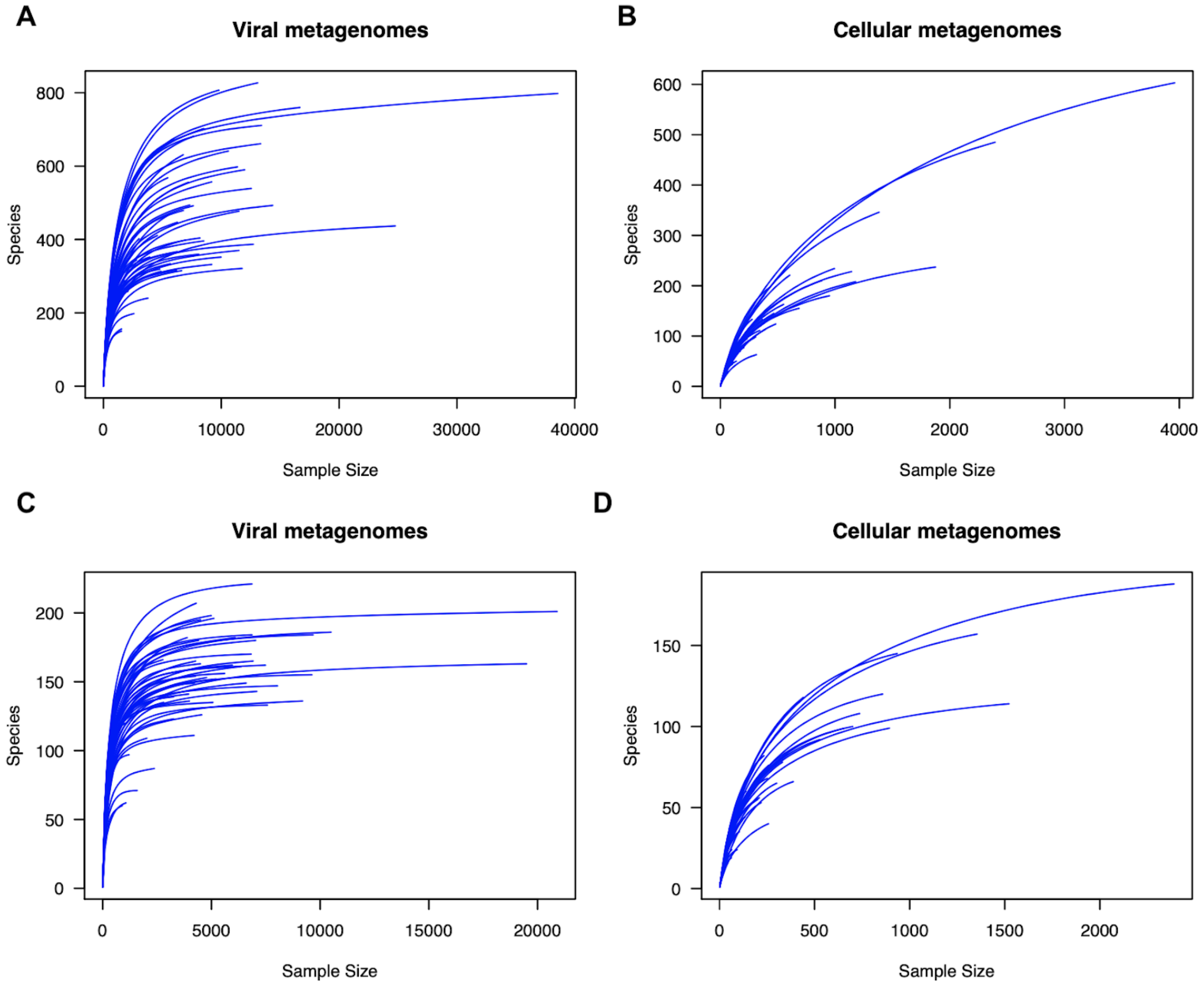

Figure S2. Rarefaction curves of *gp43* species for each viral metagenome (**A**,**C**) and cellular metagenome (**B**,**D**) using read counts for all *gp43* species detected (**A**,**C**) or for *gp43* species that were detected with 100% base coverage and 2-fold read coverage in any sample (**B**,**D**).

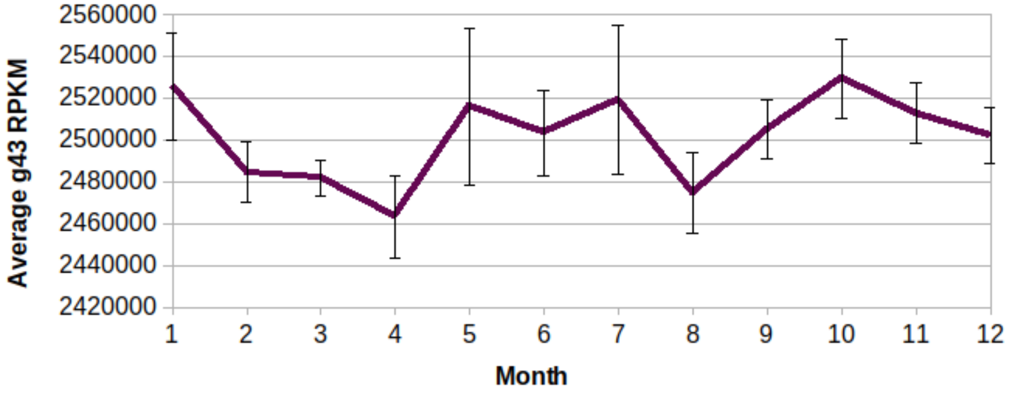

s

Figure S3. Average relative abundance of all T4-like cyanophages across the time series. Error bars are standard error.

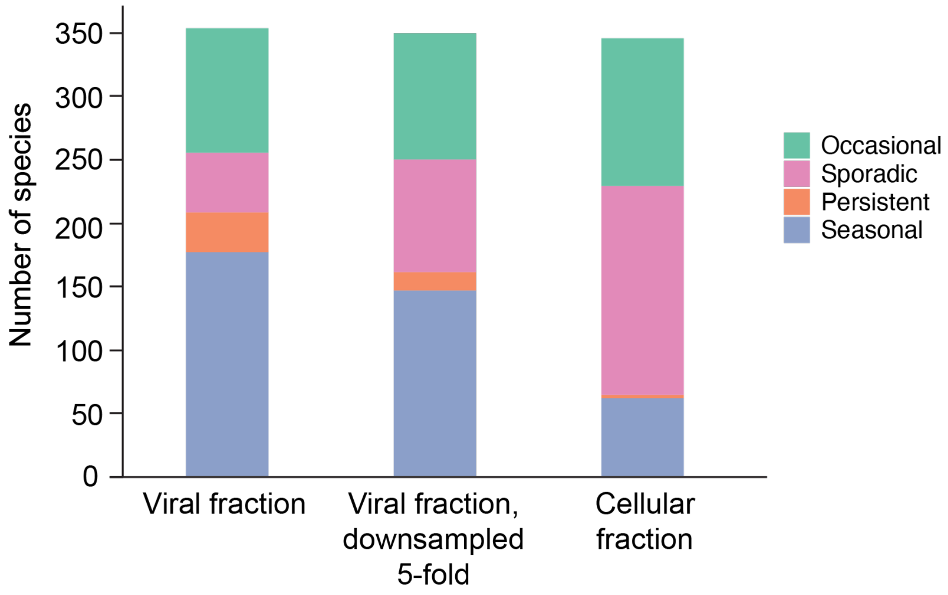

Figure S4. The number of species categorized to each dynamic class (seasonal, persistent, sporadic, and occasional) in the viral and cellular fraction metagenomes, as in Figure 3 (left and right), and when simulating 5-fold less sequencing depth for the viral fraction metagenomes (center). To simulate lower sequencing depth, read counts mapped to *gp43* species were divided by five and resulting values less than one were set to zero. Dynamic class categorization then was done as previously using resulting RPKM values (see Methods). Note that a small number of species found in the viral fraction metagenome (not downsampled) were not detected in the cellular fraction and viral fraction downsampled results, n=7 and 4 respectively.

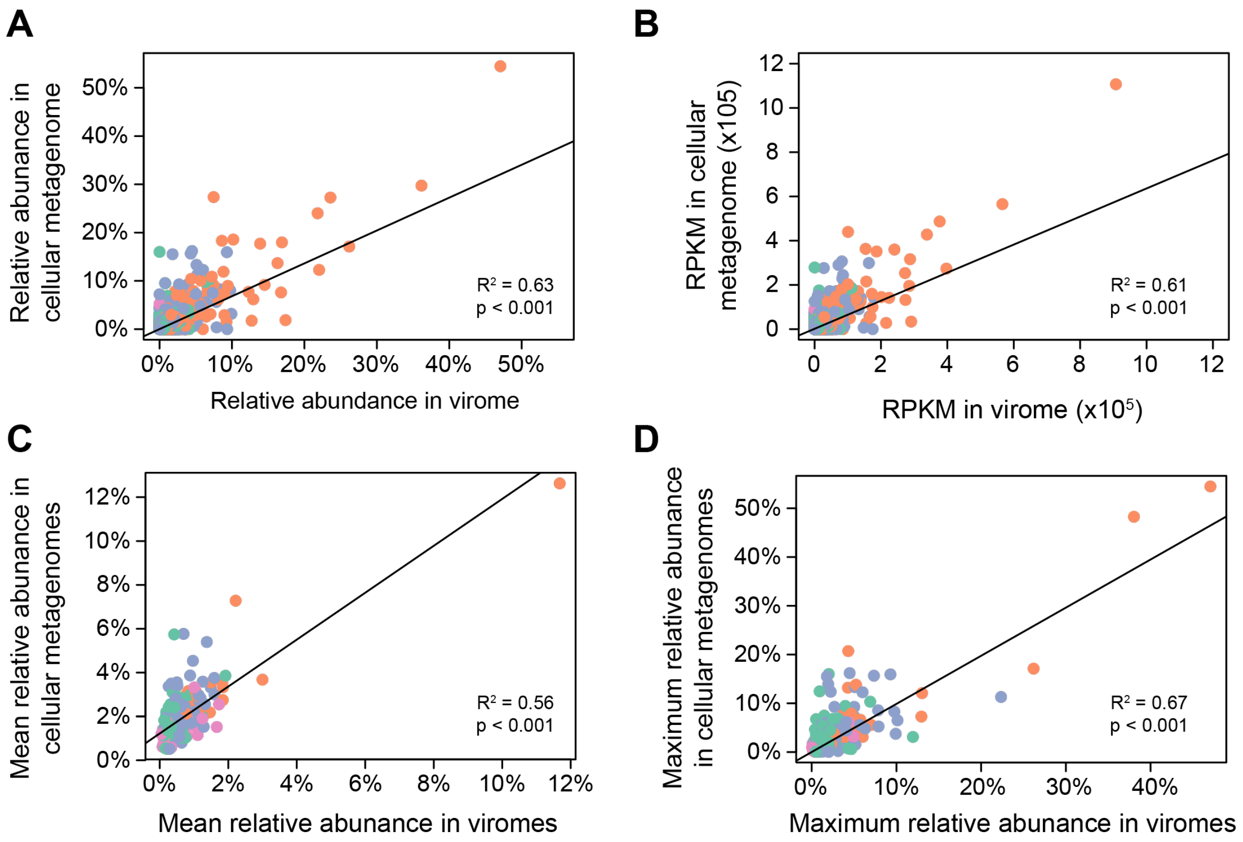

Figure S5. Scatter plots of RPKM (**A**) and relative abundance (**B**) and mean (**C**) and maximum (**D**) relative abundances of T4-like cyanophage species in the viral (x-axis) and cellular fraction metagenomes (y-axis). In **A** and **B**, each point represents the abundance for each species at each time point for months for which both viral and cellular metagenomes were available (*n*=11,427 points). In **C** and **D**, each of the points represents one of the 347 species that were detected in both the viral and cellular fraction metagenomes, and each point depicts the corresponding mean relative abundance (**A**; for months when the species was detected) or maximum relative abundance (**B**) for that species across the viral and cellular fraction metagenomes. Points are colored by their dynamic class as categorized in the viral metagenomes. Lines depict linear regressions of the data, and R^2 and p-values for the regressions are provided in the lower right of each panel.
